# Exploration of Novel Inhibitors for Class I Histone Deacetylase Isoforms by QSAR Modeling and Molecular Dynamics Simulation Assays

**DOI:** 10.1371/journal.pone.0139588

**Published:** 2015-10-02

**Authors:** Zainab Noor, Noreen Afzal, Sajid Rashid

**Affiliations:** National Center for Bioinformatics, Quaid I Azam University, Islamabad, Pakistan; Russian Academy of Sciences, Institute for Biological Instrumentation, RUSSIAN FEDERATION

## Abstract

Histone deacetylases (HDAC) are metal-dependent enzymes and considered as important targets for cell functioning. Particularly, higher expression of class I HDACs is common in the onset of multiple malignancies which results in deregulation of many target genes involved in cell growth, differentiation and survival. Although substantial attempts have been made to control the irregular functioning of HDACs by employing various inhibitors with high sensitivity towards transformed cells, limited success has been achieved in epigenetic cancer therapy. Here in this study, we used ligand-based pharmacophore and 2-dimensional quantitative structure activity relationship (QSAR) modeling approaches for targeting class I HDAC isoforms. Pharmacophore models were generated by taking into account the known IC_50_ values and experimental energy scores with extensive validations. The QSAR model having an external R^2^ value of 0.93 was employed for virtual screening of compound libraries. 10 potential lead compounds (C1-C10) were short-listed having strong binding affinities for HDACs, out of which 2 compounds (C8 and C9) were able to interact with all members of class I HDACs. The potential binding modes of HDAC2 and HDAC8 to C8 were explored through molecular dynamics simulations. Overall, bioactivity and ligand efficiency (binding energy/non-hydrogen atoms) profiles suggested that proposed hits may be more effective inhibitors for cancer therapy.

## Introduction

Histone Acetyltransferases (HATs) and HDACs regulate the acetylation and deacetylation events of small alkaline histones associated with DNA double helical structure [1,2]. Interactions of positively charged amino-terminal tails of histones with negatively charged phosphodiester backbones of DNA results in chromatin compaction [3,4]. The associated conformational changes which occur due to acetylation of lysine residues result in chromatin remodeling. Thus, HATs mediated acetylation promotes chromatin relaxation by loosening the packed histones and DNA, thereby facilitating the accession of transcription factors to bind to respective DNA templates [5–7]. In contrast to acetylation, compactness of nucleosome units and controlled gene expression as a result of deacetylation is mediated by HDACs [1]. Histones play crucial roles in stabilizing the heritable epigenetic changes in gene activity and expression [2]. Any disturbances in these functions may lead to the abnormal expression of genetic material that may cause fatal diseases like diabetes and cancer [8,9].

The critical roles of HATs and HDACs in tumor progression, cardiac and brain disorders have been analyzed in many studies [10–13]. In human, at least 18 HDACs have been identified that are grouped into four classes and comprise of two major categories: Zn^+2^ and NAD^+^ dependent [14]. This classification is based on the structural, functional and phylogenetic analysis of HDACs [15]. A detailed overview of cellular compartments and HDAC involvements in diverse biological processes is given in Table 1. Class I comprises of HDAC1-3 and 8 [16–18] which controls many functional and regulatory mechanisms [19]. Importantly, implication of class I HDACs has been monitored in hematological malignancies, resulting in differentiation and proliferation abnormalities of myeloid cells [20]. In tumor progression, most prevailing alteration is linked to abnormal expressions of HDACs [21]. Aberrant expression pattern of HDAC1 has been observed in prostate, gastric, breast and colon [22–25] cancers. Similarly, onset of gastric, cervical and colorectal carcinoma [26–28] is associated with increased expression of HDAC2. Abnormal expression patterns of class I HDAC members are also evidenced in cell proliferation and migration during ovarian and breast carcinomas [29]. In these tumor cells, down-regulation of e-cadherin is associated with HDAC3 overexpression. Unbalanced expressions of HDAC members also result in Acute Promyelocytic Leukemia (APL) such as lymphoblastic APL and non-Hodgkin’s lymphomas [28,30].

**Table 1:** Classification and biological roles of HDACs.

Classes	Cofactors	HDACs	Cellular Locations	Biological Processes	References
Class I	Zn^+2^ dependent	1, 2, 3, 8	Nucleus, Cytoplasm, Transcriptional repressor complex, Spindle microtubule, Replication fork	Cell cycle regulation, Cell differentiation, DNA damage response, Epidermis development, Regulating cardiac myocyte proliferation on the course of cardiac development	[16–18]
Class II	Zn^+2^ dependent	4, 5, 6, 7, 9, 10	Cytoplasm, Nucleus, Neuromuscular junction, Golgi apparatus, Cytosol caveola	Regulation of transcription and cell differentiation, Regulation of cardiac muscle contraction, Inflammatory response, Nervous system development, Heart development, Protein polyubiquitination, Response to toxic and organic substances, Macroautophagy, Vasculogenesis	[1, 17]
Class III	NAD^+^ dependent	Sirtuins SIRT1-SIRT7	Nucleus, Cytoplasm	Histone deacetylation, Regulation of phosphorylation, Regulation of double-strand break repair via homologous recombination, DNA repair mechanism	[16]
Class IV	Zn^+2^ dependent	11	Nucleus	Transcription, DNA-dependent chromatin modification, Histone Deacetylation	[16]

Another mechanism of HDAC-mediated tumor onset is due to transcriptional repression of tumor suppressor genes and their aberrant recruitments to promoter regions. In recent years, many studies potentiate the ways of targeted HDAC inhibition in the context of tumor control [31,32]. Transcriptional activation of tumor suppressor genes by the inhibition of HDAC activity is considered as an ideal and innovative strategy. To date, several HDAC inhibitors (HDACi) like hydroxamic acids, benzamides, short chain carbolic acids, and cyclic tetrapeptides have been characterized *in vitro* and *ex vivo* for various cancers [33–36]. Hydroxamic acids (hydroximates) include vorinostat (SAHA), belinostat (PXD101), panobinostat (LBH589), dacinostat (LAQ824), givinostat (ITF-2357) and trichostatin A (TSA). Benzamides are the derivatives of benzoic acid which include entinostat (MS-275), p-N-acetyldinaline (CI-994), mocetinostat (MGCD0103) and SK-7041. Short chain carboxylic acids (aliphatic acids) are relatively weak inhibitors which comprise of valproic acid or VPA, butyrate and sodium phenyl butyrate. Cyclic tetrapeptides or cyclic peptides are structurally complex molecules which consist of romidepsin (depsipeptide), apicidin (OSI-2040), trapoxin A and trapoxin B (cyclic hydroxamic acid) [37,38]. Currently, these inhibitors are under investigations in clinical trials [39–44]. In cancer patients, functional activity of HDACi is mediated extrinsically as well as intrinsically due to cell cycle arrest at G1/G2 phases and up-regulation of apoptosis [31,45]. Sensitivity of actinotheraphy and chemotherapy for cancers is also enhanced by HDAC inhibition [46].

*In silico* drug designing strategies are extensively applied for the identification of novel inhibitors through modeled HDAC structures and their pharmacophore-based studies [47–53]. However, these inhibitors are unique in terms of HDAC targeting actions. Here, through comparative ligand-based approaches, several novel candidate hits were proposed and added in the list of HDACi which are able to target entire class I of HDACs.

## Methodology

### Dataset

High resolution co-crystallized structures of class I HDACs were retrieved through PDB database [54] with following PDB IDs: 4BKX (HDAC1), 4LXZ (HDAC2), 4A69 (HDAC3) and 1T64 (HDAC8) (Table A in S1 File). These structures were further refined by adding charges and missing residues through *dock prep* module of UCSF Chimera 1.10 [55]. Binding and catalytic site details of class I HDACs were extracted through PDBSum [56]. Structural superimposition was performed by UCSF Chimera 1.10 [55]. Additionally, comparative binding mode of class I HDACs has been reviewed through literature [47,48,53].

### Pharmacophore/QSAR Model Generation

In order to generate the ligand-based quantitative structure-activity relationship (QSAR) and pharmacophore models, 16 known inhibitors (Table B in S1 File) were selected. These known HDAC inhibitors were collected through literature survey [57–73] and classified on the basis of their inhibition potential values for class I HDACs. Chemical structures and bioactivities of these inhibitors were extracted through EMBL-EBI ChEMBL database [74], while their 2D/3D molecular descriptors values were retrieved through ChemAxon’s Chemicalize [75] and Molinspiration [76]. Molecular dockings of these inhibitors were performed against class I HDACs by AutoDock VINA with default parameters i.e. exhaustiveness = 8 and energy range = 3 [77].

The selection of training set compounds was based on bioactivity and binding affinity attributes of docked protein-inhibitor complexes. The best docked conformations of inhibitors having least IC_50_ values and high binding abilities were filtered out for pharmacophore generation. In order to design 2D QSAR model, IC_50_ values of training set compounds against HDAC1, 2, 3 and 8 were converted into pIC_50_ values and average pIC_50_ values were calculated for each of the training set compound.

Test data set was created to evaluate the quality of pharmacophore hypotheses. Known selective and non-selective synthetic molecules were gathered through ChEMBL [74] which were utilized in human assays [68,69,71,73,78–109] to generate a dataset of 71 compounds (Table C in S1 File) and their experimental IC_50_ values were assessed. Compounds having weak inhibition potential (IC_50_ > 2500 nM) for class I HDACs were grouped in decoy set, whereas high inhibitory compounds (IC_50_ < 2500 nM) were placed in the active dataset. By implying the above criteria, 41 compounds were found to be active and 30 were declared as decoys. 3D generation and optimization of data set compounds was performed by ACD ChemSketch [110].

To generate pharmacophore models, training set hits were employed in the ligand-based module of the LigandScout 3.0 [111]. Pharmacophoric sites such as hydrogen bond donor (HBD), hydrogen bond acceptor (HBA), hydrophobic sites, aromatic ring and positive and negative ionizable groups were carefully characterized. To incorporate the associated features of selected compounds, merge feature model generation and atom overlap scoring function of LigandScout 3.0 was applied. Subsequently, descriptor selection analysis was performed through forward selection (FS), backward elimination (BE) and stepwise selection (SS), the classical methods of variable selection [112]. All unique descriptors signifying the model were selected, while excluding the non-significant descriptors. QSAR model was created by employing multiple linear regression (MLR) technique [113]. Regression module of the IBM statistical package for social sciences (SPSS) version 22 [114] was used to create the regression model by employing Eq 1.

$$y_{i}=\;\beta_{0}+\beta_{1}x_{i}+\varepsilon_{i},\;i=1,\ldots .,n$$ (1)

Where, *y* (pIC_50_) is the dependent variable, *x* is the independent variable, *β* is the coefficient and *ε* is the error value. QSAR and pharmacophore models were statistically evaluated to check their reliabilities prior to implementation.

Top five models exhibiting significant statistical values were scrutinized as 3D query for virtual screening. Validated 3D pharmacophore models were screened against a total of 48,386 compounds (2,601 of Aurora [115] and 45,785 of Princeton [116] libraries) to prey novel drug targets. The novel hits were selected for docking analysis on the basis of high query fit values relative to pharmacophore models.

### Molecular docking and dynamic simulation assays

Molecular docking analyses of highly ranked compounds were performed for class I HDACs through above mentioned parameters of AutoDock VINA [77]. Subsequently, energy scores and HDAC bound poses of these compounds were carefully evaluated. To study the structural dynamics of receptor-ligand complex, molecular dynamics (MD) simulations of HDAC2 and HDAC8 were performed. Gromacs 4.5.5 [117] and GROMOS96 43a1 force field with SPC water model was used for MD simulations. Subsequently, energy minimization was performed by steepest descent method and appropriate counter ions were added to neutralize the system [118,119]. Finally, a 12 ns MD simulation run was performed at constant temperature (300 K) and pressure (1 atm). GROMACS analysis tools were used for the analysis of MD simulation trajectories (118). The stability of secondary structure elements, conformational changes and interactions were assessed by computing root mean square deviation (RMSD), root mean square fluctuation (RMSF) and hydrogen bonds obtained throughout MD trajectories. For RMSD, RMSF, gyration and hydrogen bonds g_rms, g_rmsf, g_gyrate and g_hbond modules of Gromacs were applied, respectively. RMS cluster distribution of HDAC2 and HDAC8 backbone was computed using g_cluster module with nearest neighbor method. Energy values along the MD trajectories were calculated by Eq 2.

$$E=\;E_{bond\;stretch}+\;E_{angle\;bend}+\;E_{rotation}+\;E_{vander\;waals}+\;E_{electrostatic}$$ (2)

All simulations were carried out through an open SUSE 11.2 system with Intel(R) core (TM) i5-2300 CPU containing Linux 2.6.31.5–0.1 operating system. The binding patterns were carefully monitored through Accelrys Discovery Studio 4 [120].

ADMET (Absorption, Distribution, Metabolism, Excretion and Toxicity) properties were predicted through AdmetSAR [121] server to hypothetically depict the positive and negative biological effects of compounds. Bioavailability, Rule of Five (ROF), lead-likeness and other filters were assessed using Chemicalize [75]. Toxicity parameters such as mutagenic and tumorigenic effects of selected compounds were evaluated through OSIRIS [122].

Expected IC_50_ values of compounds were predicted through QSAR model. Physiochemical descriptors of compounds were extracted by Chemicalize [75] and Molinspiration [76] servers. To avoid any redundancy issue, predicted compounds were compared against small molecule library available in ChEMBL [74] database, based on ≥90% similarity threshold. Synthetic accessibility analysis of predicted hits was performed through SYLVIA [123] tool.

## Results

### Binding site analysis

**Fig 1:**
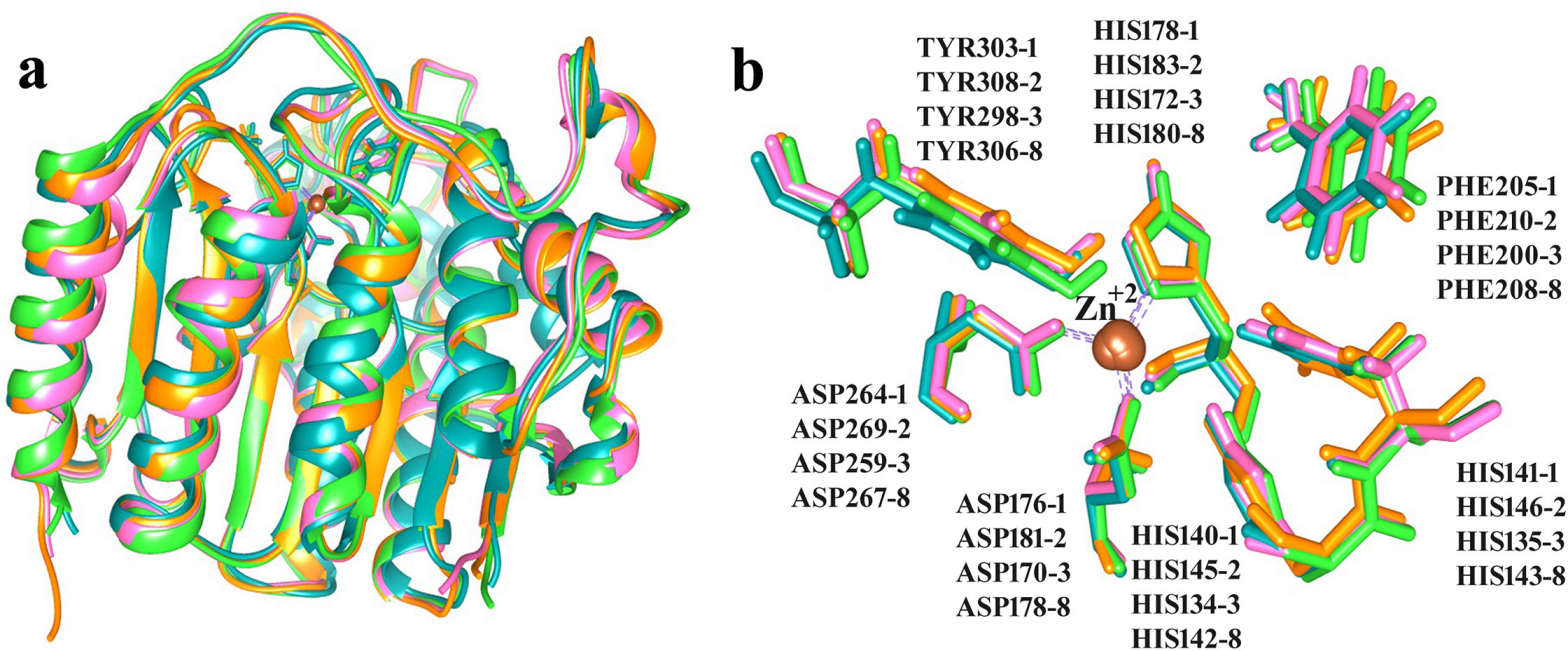
Zn^+2^ dependent class I HDACs. **(a)** Structural superimposition of HDACs. **(b)** Binding sites of class 1 HDACs. HDAC1, 2, 3 and 8 are shown in orange, pink, green and blue, respectively. Zn^+2^ is shown in brown color.

Binding sites of class I HDAC family members were evaluated by structural comparisons (Fig 1). Co-crystallized structures of HDAC1, 2, 3 and 8 showed structural similarities with an RMSD value of 0.5Å (Fig 1A). Zn^+2^ ions were coordinated by one HIS and two ASP residues of individual HDACs (Fig 1B). These catalytic residues resided within the 4Å region of surface forming the tunnel shaped pocket.

### Pharmacophore-based 2D QSAR modeling

In order to select training set compounds, binding affinities, IC_50_ and molecular descriptors values of known class I HDAC inhibitors were monitored (Tables B, D and E in S1 File). These known hits included MS-275, LBH-589, LAQ-824, Trichostatin A, Saha, Belinostat, Oxamflatin, Pyroxamide, Mocetinostat, and Scriptaid with IC_50_ values ranging from 5–300 nM. 5–7 pharmacophore models were generated based on the information of docking poses of training set compounds and their pharmacophore fit values were evaluated. The ideal pharmacophore models with high query fit values were shown in Figure A in S1 File. These models exhibited good pharmacophoric features such as hydrogen bond donors, hydrogen bond acceptors, hydrophobic sites and negative ionizable groups (Zn^+2^ binding locations). QSAR regression model was generated using 5 descriptors of training set compounds, as shown in Eq 3. Their pIC_50_ values were shown in Table F in S1 File.

$$pIC_{50}=\;14.48\left(\pm 3.8\right)-0.13\left(\pm 0.06\right)*Mol.polarizability+0.06\left(\pm 0.04\right)*logP+\;0.17\left(\pm 0.04\right)*Topological\;Polar\;Surface\;Area\;\left(TPSA\right)-2.54\left(\pm 0.5\right)*Hy.Bond\;Acceptor-2.57\left(\pm 1.21\right)*Balaban\;Index$$ (3)

**Fig 2:**
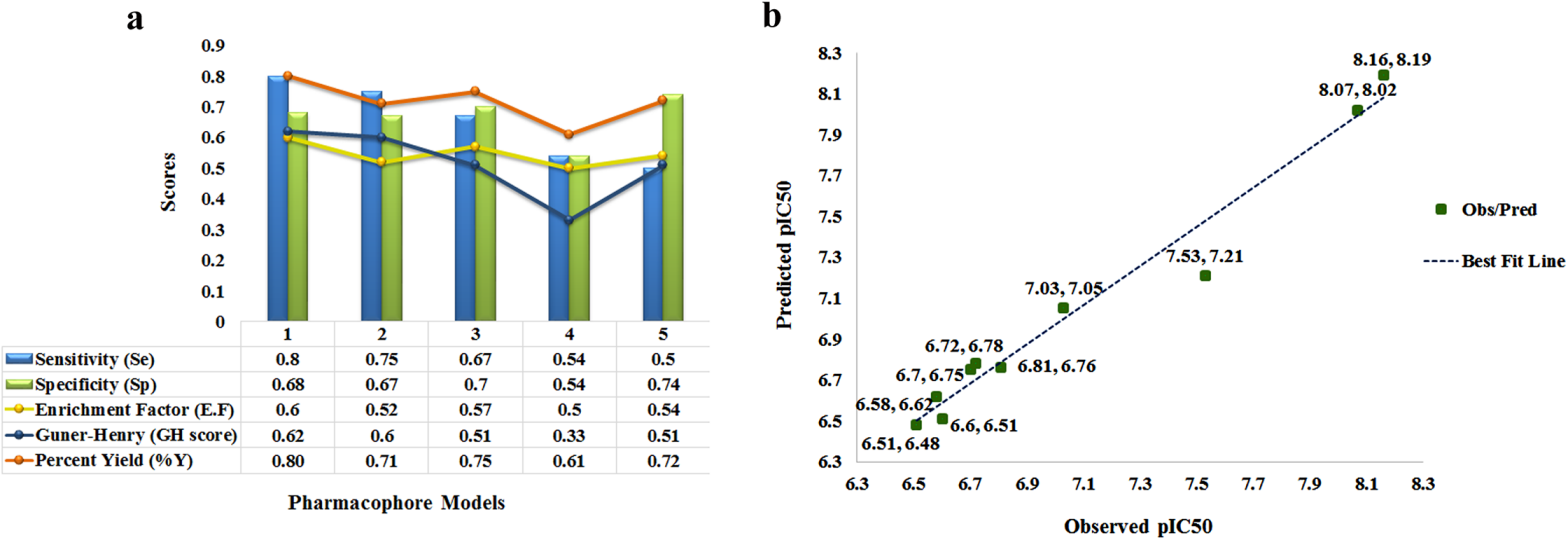
Statistical validation of designed pharmacophore and QSAR models. **(a)** Top five pharmacophore models labeled as Model (1–5). **(b)** Correlation analysis of QSAR regression model. Green dots depict the observed vs predicted IC_50_ values and correlation best fit dotted line is shown in blue.

To determine the reliability and predictability of models, several statistical parameters (Table G in S1 File) were applied which showed positive results. Higher sensitivity and specificity values, percent of active yield (%Y), enrichment factor and Guner-Henry score depict the performance quality of pharmacophore model [124] (Fig 2A). Our generated models showed high sensitivity and specificity values; models 1 and 2 resulted in 80% and 75% retrieval of active compounds, respectively. Scores of other statistical parameters were comprised of 80% score of number of active percent of yields (%Y) and G.H scores of 0.7 and 0.65 for models 1 and 2, respectively. Statistical evaluation of QSAR model included goodness of fit i.e. R^2^ (correlation coefficient) and adjusted R^2^ (goodness of fit) values (coefficient of determination). The R^2^ value (0.93) and adjusted R^2^ (0.84) were close to 1 and standard error value was close to 0 (Table 2). External validation was performed by predicting pIC_50_ values of training set compounds through generated model and cross-validated against the given activity values (Fig 2B). Observed IC_50_ values of training set compounds were comparable with the predicted values and residual error values were ≤ 0.5. ANalysis of VAriance (ANoVA) test [125] also verified the model with a p-value of 0.02.

**Table 2:** Statistical parameters for regression model.

N	R	R^2^	Adjusted R^2^	Std. Error	Significance
10	0.96	0.93	0.84	0.24	0.02

### Virtual screening

**Fig 3:**
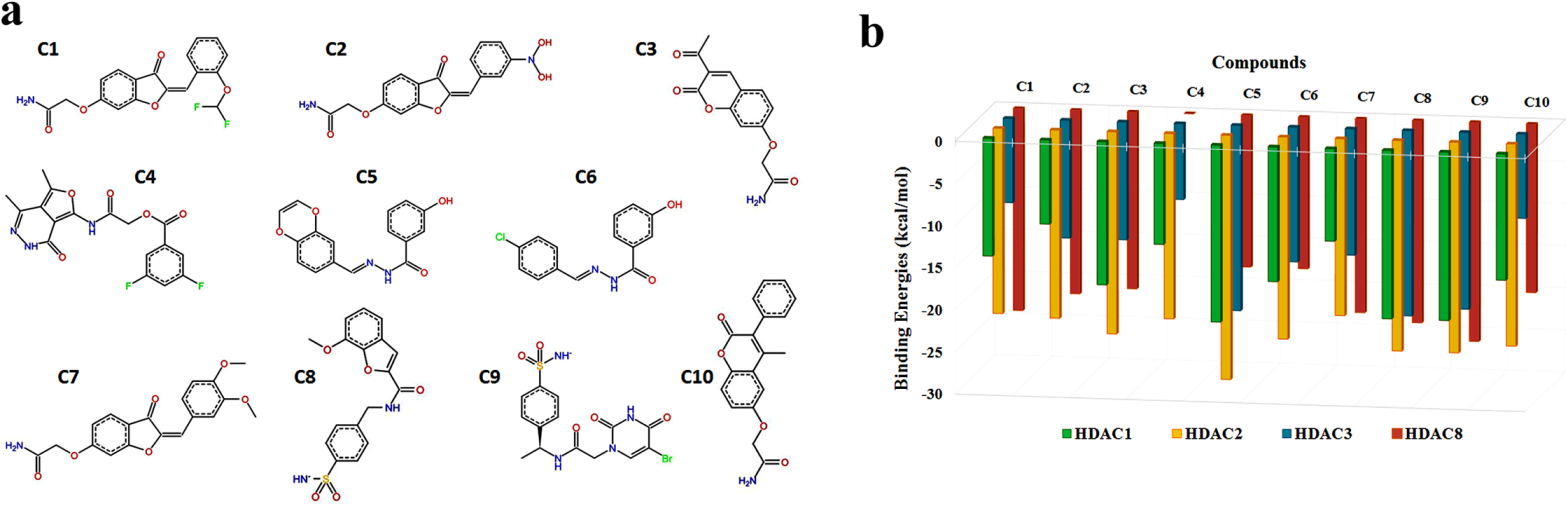
2D structures and binding energies of compounds C1-C10. **(a)** 2D structures **(b)** Binding energy overview.

**Fig 4:**
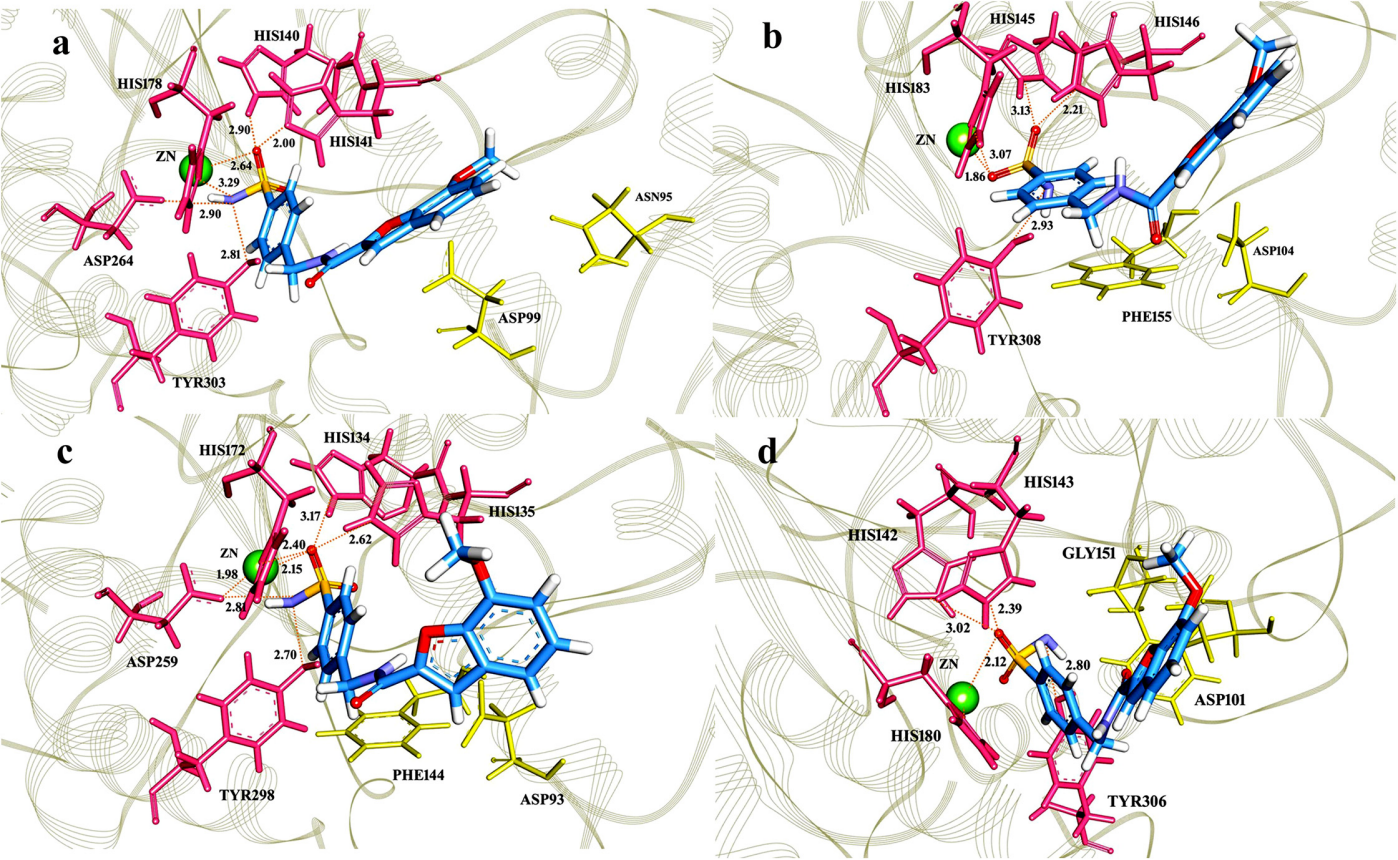
Binding pattern of compound C8 with class 1 HDACs. C8 (7-methoxy-N-((4-sulfamoylphenyl)methyl)-1-benzofuran-2-carboxamide) was shown in blue, whereas hydrogen bonding and hydrophobic residues were shown in pink and yellow, respectively. **(a)** Hit C8 forms hydrogen bonds with HIS140, HIS148, HIS178, ASP264 and TYR303 of HDAC1; **(b)** HIS145, HIS146, HIS183 and TYR308 of HDAC2; **(c)** HIS134, HIS135, HIS172, ASP259 and **(d)**TYR298 of HDAC3 and HIS142, HIS143, HIS180 and TYR306 of HDAC8. C8 bonding with Zn+2 is shown in green color with an average distance of 2Å. Hydrophobic residues involved in interaction are **(a)** ASP99, GLY149, PHE150 and GLY301 in HDAC1; **(b)** PHE155, PHE210, ASP269 and GLY306 in HDAC2; **(c)** ASP93, PHE144, ASP170, PHE209 and GLY296 in HDAC3 and **(d)** PHE152, PHE208, MET274 and GLY304 in HDAC8.

Pharmacophore model was incorporated to the virtual screening of compound libraries and about 2000 hits were screened out with high to low query fit values. 81 top scoring hits were selected for docking and drug-like analysis. 16 novel hits (1–16 in Table H in S1 File) exhibited interactions with specified binding sites of class I HDACs. These novel compounds were further exploited for the analysis of steric and physiochemical properties (Table I in S1 File) and 10 compounds (C1-C10) were identified as potential inhibitors for class I HDACs. The 2D structures and binding profile of these compounds are listed in Fig 3 and Table J in S1 File indicates their IUPAC names and SMILE codes. These compounds (C1-C10) exhibited interactions with Zn^+2^ and binding pocket residues of class I HDACs. C8 and C9 showed class specific binding (Fig 4), while other hits were specific for individual HDACs (Figures B-E in S1 File). Binding pattern of new drug-like compounds coincides with the experimental known binding of existing inhibitors. Bound complexes of SAHA with HDAC2 and Trichostatin A with HDAC8 were shown in Figure F in S1 File which depicted that these inhibitors have similar interaction mode as described for the newly identified inhibitors (Figures B-E in S1 File). Interacting residues and distances of proposed compounds (C1-C10) were also in good agreement to the experimental results.

**Fig 5:**
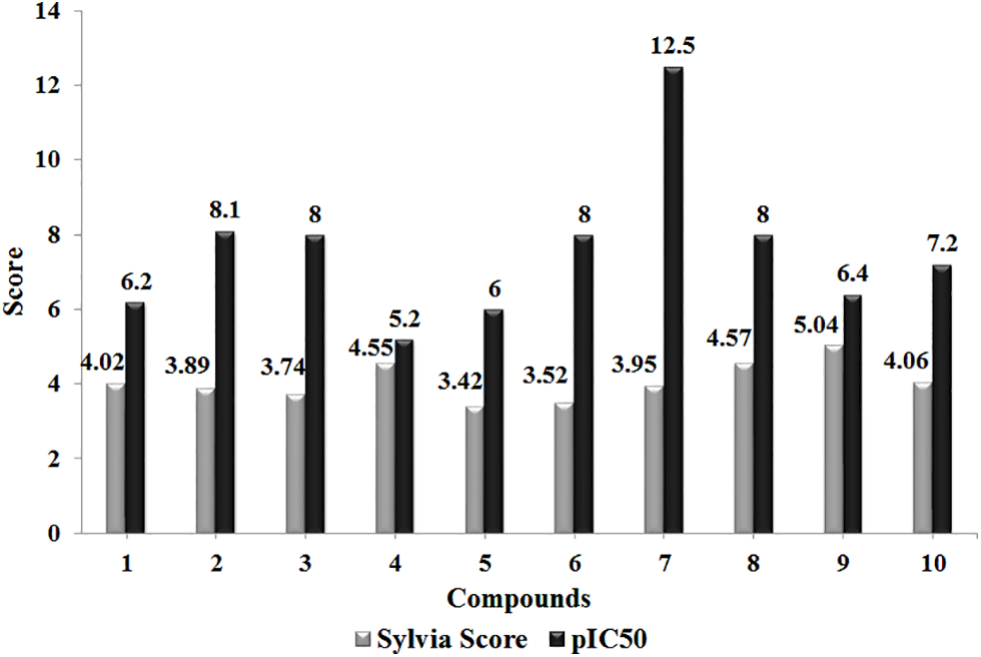
Synthetic accessibility score and expected pIC_50_ values.

ADMET analysis, binding contributions and physiochemical properties suggested that predicted hits (C1-C10) (Table 3) may act as more potent inhibitors against class I HDACs. Synthetic accessibility scores (<5) of these hits (Fig 5) further validated these hits. The expected pIC_50_ values of these hits were predicted by QSAR model (Eq 1) and compared with the known pIC_50_ values of training set compounds. As evident, hit C7 (pIC_50_ = 12.5) is the most bioactive compound (Fig 5).

**Table 3:** ADME and Toxicity Analysis.

Compounds	Human Intestinal Absorption	CYP450 Inhibition	Blood Brain Barrier	Bioavailability	No Mutagenic	No Tumorigenic	No Irritant
C1	1	0.72	0.964	+	+	+	+
C2	0.8	0.6	0.7	+	+	+	+
C3	0.9	0.8	0.7	+	+	+	+
C4	0.9	0.6	0.7	+	+	+	+
C5	0.7	0.6	0.7	+	+	+	+
C6	0.9	0.8	0.9	+	+	+	+
C7	1	0.8	0.7	+	+	+	+
C8	1	0.8	0.8	+	+	+	+
C9	0.9	0.8	0.6	+	+	+	+
C10	1	0.7	0.6	+	+	+	+

Recently, the properties like ligand efficiency (LE) or *binding energy of ligand per atom* [126–128] and lipophilic efficiency (LipE or LLE) [129, 130] have been considered essential for lead optimization [131,132], which rely on both potency and lipophilicity profiles. LE is the ratio of free energy of binding to the number of heavy atoms and was calculated by assuming the standard conditions of aqueous solution i.e. 300K, neutral pH and remaining concentrations of 1M (Eq 4) [133]. To access the lipophilicity of predicted hits, logP (activity/size) values were calculated (Table 4) using Bio-Loom version 1.5 [134] and compared with pIC_50_ values. LE (Eq 4) [135,133] and LipE (Eq 5) [129] profiles of inhibitors were used to identify the hits with higher activities (Table 4).

**Table 4:** Ligand efficiency (LE), Lipophilic Efficiency (LipE) and Fit Quality (FQ) values of C1-C10 hits.

Compounds	pIC_50_	HA	clogP	LE	LEScale	LipE	FQ
C1	6.2	25	2.94	0.34	0.36	3.26	0.95
C2	8.1	25	0.81	0.44	0.36	7.29	1.22
C3	8	19	0.27	0.58	0.43	7.73	1.35
C4	5.2	27	1.49	0.26	0.34	3.71	0.76
C5	6	22	4.11	0.37	0.29	1.89	1.28
C6	8	19	3.38	0.58	0.43	4.62	1.35
C7	12.5	26	2.23	0.66	0.35	10.27	1.89
C8	8	25	1.7	0.44	0.36	6.3	1.22
C9	6.4	25	-0.2	0.35	0.36	6.6	0.97
C10	7.2	23	2.5	0.43	0.38	4.7	1.13

$$LE\;=\left(1.37/HA\right)*\;pIC_{50}$$ (4)

LLE=pIC50−clogP (5)

Size-independent ligand efficiency values (LEScale) of these hits were calculated through fitting the top LE values with heavy atom count through a simple exponential function (Eq 6), as described [127,128]. “Fit Quality” or “FQ” scoring function (Eq 7) is the ratio of LE and LEScale which was computed to detect the optimal ligand binding properties of predicted hits.

$$LEScale\;=0.104+0.65e^{-0.037*HA}$$ (6)

FQ=LE/LEScale (7)

**Fig 6:**
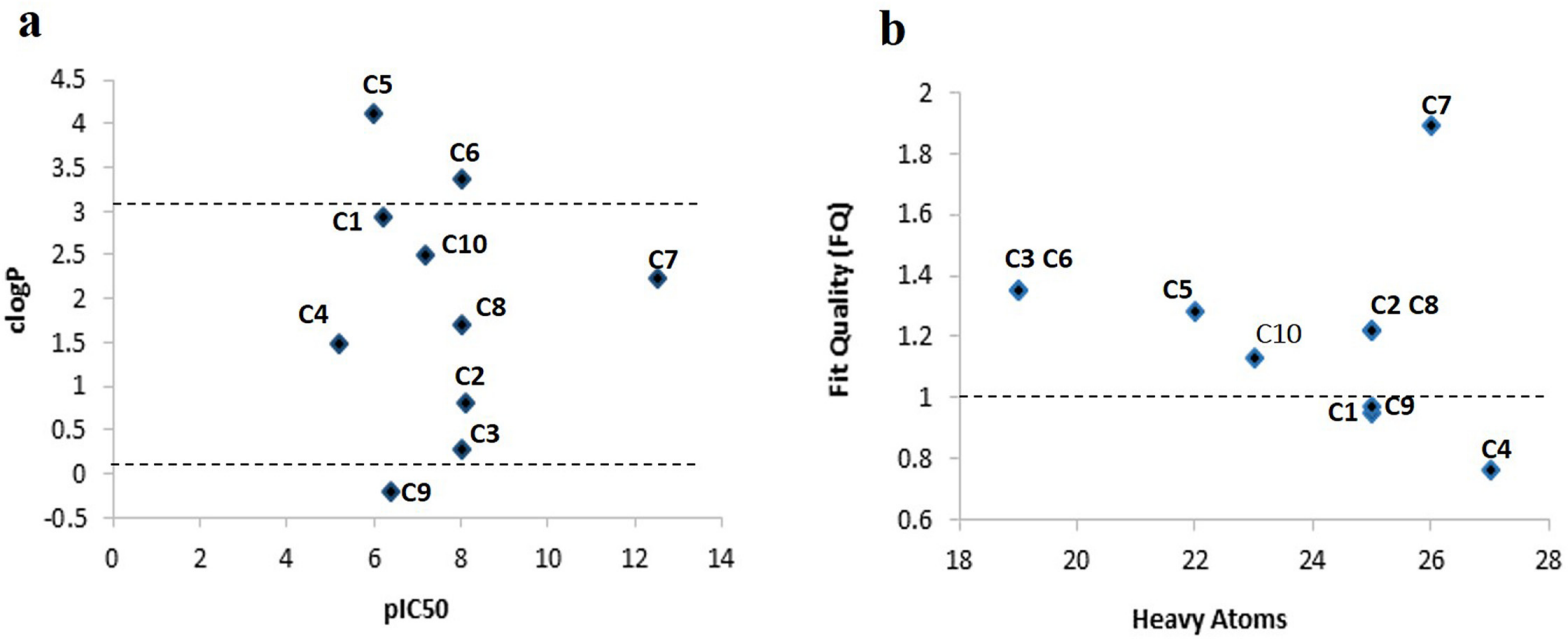
Ligand efficiency analysis of 10 selected compounds (C1-C10). (a) ClogP and (b) Fit Quality graph.

Conventionally, clogP <3, LipE > 5 and FQ close to 1 are considered as optimal properties for highly bioactive compounds [127–129]. Clearly, clogP values of C1, C2, C3, C4, C7, C8 and C10 hits ranged in 0.2 to 2.5 (Fig 6A), while about 50% compounds exhibited LipE > 5 (Table 4). Similarly, FQ scores of these compounds were also in acceptable range (Fig 6B), indicating that proposed hits may have better *in vivo* performance based on their ligand binding, potency and lipophilicity profiles.

### Molecular dynamics simulation analysis

**Fig 7:**
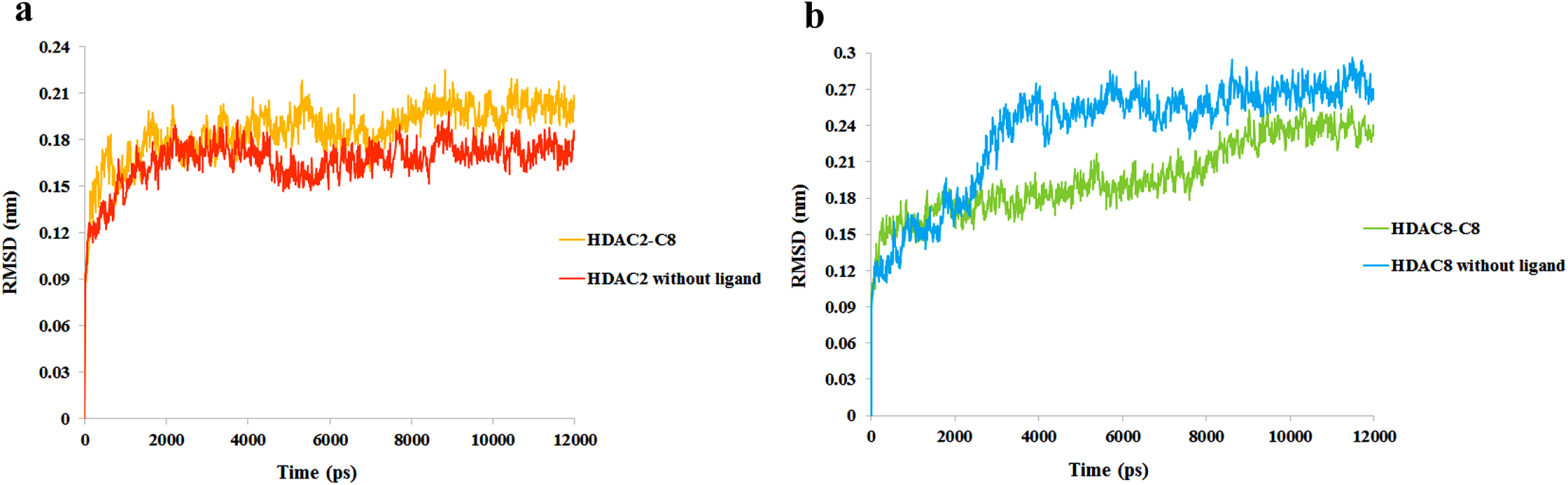
RMSD plot for 12 ns MD simulation. (a) RMSD plot for HDAC2-C8 complex (yellow) and HDAC2 without ligand (red). (b) RMSD plot for HDAC8-C8 complex (green) and HDAC8 without ligand (blue).

To elucidate the dynamic behavior of class I HDACs upon binding to inhibitor and to gauge the pattern of system stability, HDAC2 and HDAC8 were subjected to molecular dynamics (MD) simulations. The stability of secondary structure elements and conformational changes were assessed by computing root mean square deviation (RMSD) and root mean square fluctuation (RMSF) plots of values obtained throughout MD trajectories. RMSD is a measure of stable interaction pattern of docked complex. In case of HDAC2, RMSD trend remained stable for both bound and unbound complexes within the range of 1.5-2Å (Fig 7A). However, In case of apo HDAC8, an increasing trend of RMSD profile (1.5Å to 2.8Å) was observed between 2–4 ns, while in its bound form with C8, system attained lower RMSD values in a range of 1.5Å- 2.2Å (Fig 7B). Our analysis indicated that backbone RMSD profiles of HDAC2 and HDAC8 systems remained stable during 12 ns MD runs.

**Fig 8:**
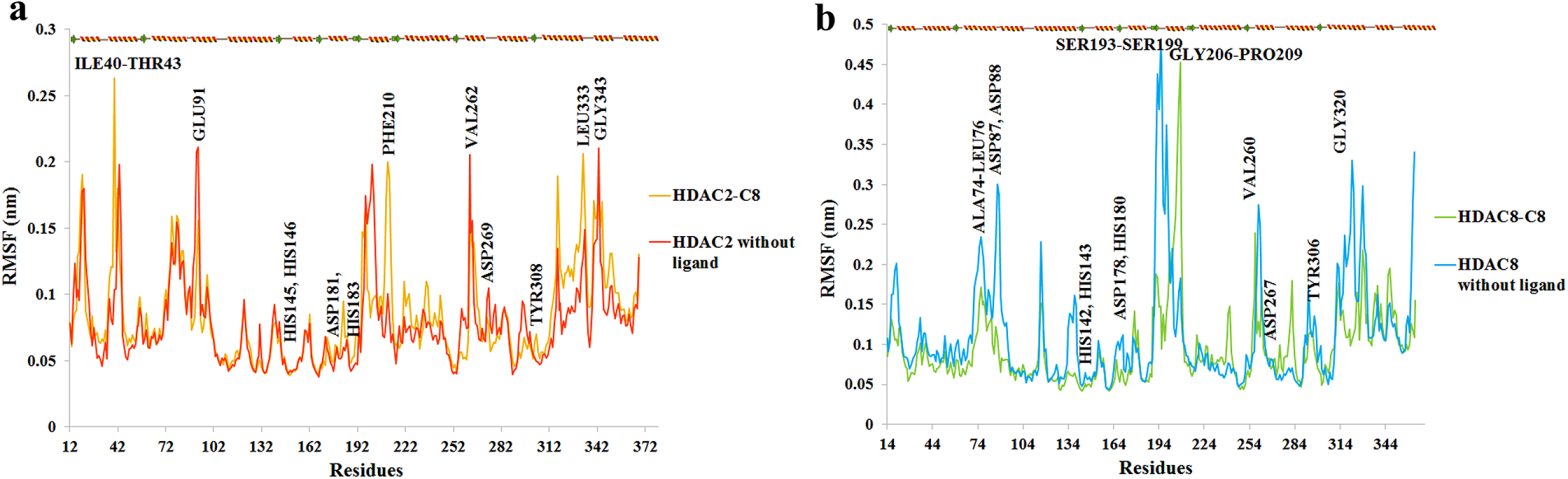
RMSF plot for 12 ns MD simulation. (a) RMSF plot for HDAC2-C8 complex (yellow) and HDAC2 without ligand (red). (b) RMSF plot for HDAC8-C8 complex (green) and HDAC8 without ligand (blue).

RMSF plots provided insight into the residual fluctuations upon binding to inhibitor (Fig 8). In HDAC2 bound to C8, higher RMSF values were observed for ILE40-THR43 (2–2.7Å), PHE210 (0.8-2Å) and LEU333 (1.5-2Å) residues (Fig 8A), while residues involved in inhibitor binding namely, HIS145, HIS146, ASP181, HIS183, ASP269 and TYR308 were quite stable. In HDAC8-C8 system, fluctuations were more common in ASP87, ASP88, SER193-SER199 and GLY320 residues. However, RMSF profiles of binding site and Zn^+2^ coordinated residues (HIS142, HIS143, ASP178, HIS180, ASP267 and TYR306) exhibited a lower trend (Fig 8B). The observed conformational changes occurring in the proximal residues of HDAC2 and HDAC8 binding sites induced more flexibility to accommodate the inhibitor.

**Fig 9:**
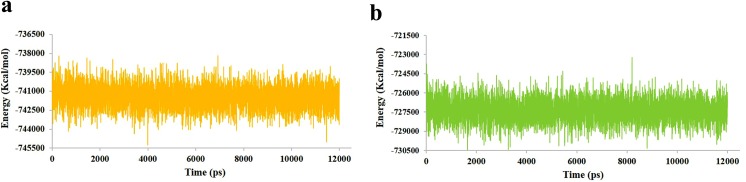
Energy plot for 12 ns MD simulation. (a) Energy plot for HDAC2-C8 complex (yellow). (b) Energy plot for HDAC8-C8 complex (green).

**Fig 10:**
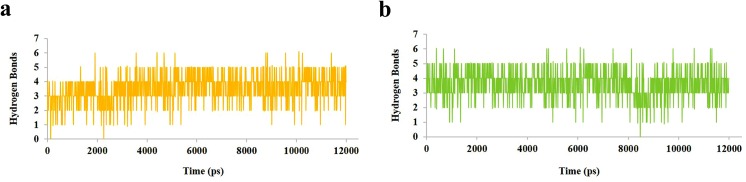
Hydrogen bonds plot for 12 ns MD simulation. (a) Hydrogen bonds for HDAC2-C8 complex (yellow). (b) Hydrogen bonds for HDAC8-C8 complex (green).

**Fig 11:**
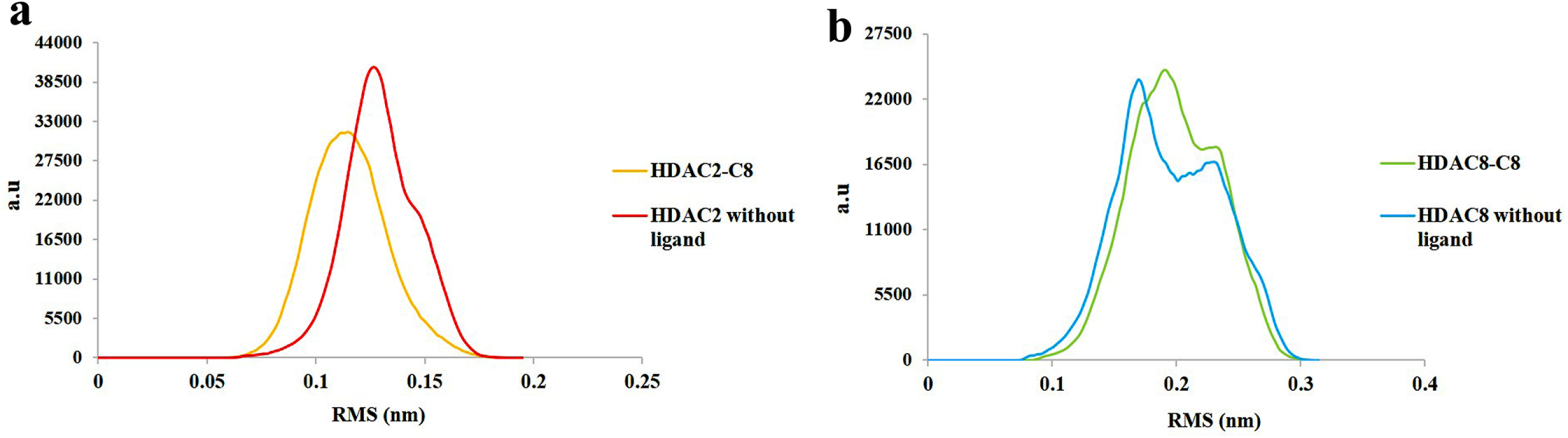
RMS distribution for 12 ns MD simulation. (a) RMS distribution pattern for HDAC2-C8 complex (yellow) and HDAC2 without ligand (red). (b) RMS distribution for HDAC8-C8 complex (green) and HDAC8 without ligand (blue).

**Fig 12:**
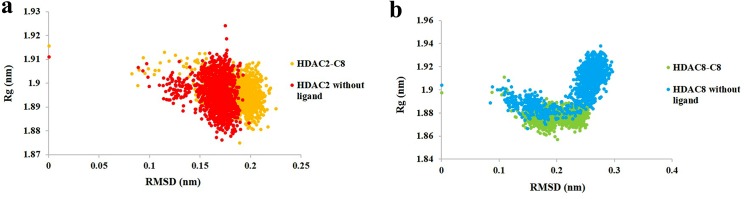
Radius of gyration (Rg) analysis for 12 ns MD simulation. (a) Rg/RMSD plot for HDAC2-C8 complex (yellow) and HDAC2 without ligand (red). (b) Rg/RMSD for HDAC8-C8 complex (green) and HDAC8 without ligand (blue).

The potential energy of system is a measure of its stability. By plotting potential energy as a function of time, we observed that systems were well equilibrated and remained stable throughout MD simulations. The HDAC2-C8 (-74100 kcal/mol) system has shown lower potential energy values compared to HDAC8-C8 (-727500 kcal/mol) complex (Fig 9). The binding characteristics of HDAC2 and HDAC8 with C8 were analyzed through plotting time-dependent intermolecular hydrogen bonds. Compared to HDAC8-C8 system, the intermolecular hydrogen bonds were increased in HDAC2-C8 complex after 2 ns of simulation time, indicating higher interactions (Fig 10). Overall, hydrogen bond interactions remained stable throughout the simulation time. These results substantiated that C8 exhibited more stable binding to HDAC2 compared to HDAC8, which is in good agreement to the distribution of RMSD distances of trajectory conformation pairs. The lower mean RMS values in case of HDAC2-C8 complex displayed the stable nature of system compared to apo HDAC2, while in C8 bound and unbound HDAC8, the pattern of RMS distribution was quite similar (Fig 11). Analysis of radius of gyration (Rg) together with RMSD profile revealed a convergence of Rg values between 1.88 nm to 1.91 nm for HDAC2-C8 complex (Fig 12A). In case of apo HDAC8, Rg trend was quite different compared to its bound form (Fig 12B). These data indicated that structural transitions in HDAC2 resulted in less tight packing, while in HDAC8, binding of C8 induced more compaction in the structure.

## Discussion

Ligand-based pharmacophore modeling, based on the energetic binding values of inhibitors (e-pharmacophore) is considered as effective tool in rational drug design [53]. Here we retrieved a diverse set of inhibitors for class I HDACs by generating pharmacophore/QSAR models based on QSAR properties of MS-275, LBH-589, LAQ-824, Trichostatin A, Saha, Belinostat, Oxamflatin, Pyroxamide, Mocetinostat, and Scriptaid. The quality of our selected model (R^2^ value of 0.93) was quite high, compared to previously proposed pharmacophore/QSAR models for screening HDAC inhibitors [49–51]. Subsequently, 10 novel hit compounds (C1-C10) were determined through their query fit values and pharmacophoric features they possess. Our current protocol of pharmacophore/QSAR-based virtual screening was quite efficient in predicting the reliable inhibitors as it was based on a subset of selective descriptors which were based on known scaffolds.

Through docking analysis, the candidate hits with binding affinities for all members of class I HDACs were selected for detailed analysis. The structural features of predicted hits were much similar by having a capping region, a linker and a Zn^+2^ ion binding region. The predicted hits were derivatives of benzamides, acetamides, carboxamides and hydrazides. Out of these hits, C8 (7-methoxy-N-[(4-sulfamoylphenyl)methyl]-1-benzofuran-2-carboxamide) and C9 2-(5-bromo-2,4-dioxo-1,2,3,4-tetrahydropyrimidin-1-yl)-N-[1-(4-sulfamoylphenyl)ethyl]acetamide showed ideal binding energetics for HDAC1, 2, 3 and 8 (Fig 3). In C8, carboxamide moiety was involved in hydrogen bonding with Zn^+2^ metal ion as well as other active site residues. Due to possessing Zn^+2^-binding amide moieties and stereo selectivity, carboxamides are considered as more potent inhibitors for HDACs [136,137]. Recent experimental evidences support benzofuran-2-carboxamide derivatives for their antitumor and anti-proliferative properties [138]. Similarly, various benzofuran-2-carboxylic acids bearing (chlorometyl) indoline or benzoyl nitrogen as DNA-binding group serve as structural subunits of synthetic analogues of natural antitumor agents such as dystamycin, CC-1065, duocarmycin, and netropsin [139–141]. They also act as adenosine A2A receptor antagonists [142]. Based on the essential value of carboxamides, hit C8 was analyzed in detail for its binding to class I HDAC isoforms. In the catalytic pockets of HDACs, sulfamoylphenyl ring of C8 actively participated in the hydrogen bonding with HIS140, HIS148, HIS178, ASP264 and TYR303 residues of HDAC1; HIS145, HIS146, HIS183 and TYR308 of HDAC2; HIS134, HIS135, HIS172, ASP259 and TYR298 of HDAC3 and HIS142, HIS143, HIS180 and TYR306 of HDAC8 (Fig 4). These interactions were mainly mediated by the conformational readjustments of linker region, while benzofuran ring was uniquely involved in hydrophobic interactions. Consequently, the O-atoms of SO_2_ coordinated with metal ion in HDAC hydrophobic pockets. Indeed, study of hydrogen bond network between catalytic metal ion and sulfonamide group may be useful to synthesize more potent inhibitors for HDACs.

The proposed aromatic/heterocyclic compounds possessed isoform-specific interactions. For example, crystal structure of HDAC1 exhibited binding with C3, C5 and C6 hits, HDAC2 showed binding with C1-C10, HDAC3 with C5, C6 and C7, while HDAC8 was complexed to C1, C2, C3, C7 and C10. Interestingly, C1, C4, C6 and C9 compounds exhibited halogens as capping groups which indicated that these inhibitors may prefer class I HDACs for binding. The importance of halogenated capping in the selectivity of inhibitors has been well documented. In a series of aroyl pyrrolyl hydroxamide (APHA) compounds screened against maize HD1-B and HD1-A (homologues of mammalian class I and class II HDACs), non-halogenated derivatives showed no selectivity for class I or II [143]. Modification of the capping group region led to 176-fold selectivity for class I HDAC over II. These data underscore that halogens impart class selectivity. However, further analysis is warranted to understand the significance of capping region in parallel to variations in the choice of linker region and metal binding groups in HDACi selectivity.

Moreover, compounds with conserved scaffolds exhibited the comparable binding pattern and energetics. Here, C1, C2 and C7 analogues (group I) possessed similar linker and Zn^+2^ ion binding regions with distinguished capping region substituents i.e. difluoromethoxy, carbamoylmethoxy and dimethoxyphenyl, respectively (Fig 3A). Likewise, compounds C5 and C6 (group II) were distinct only in the capping site where the benzodioxin in C5 was replaced by chlorophenyl in C6. (Fig 3A). Energy profiles of group I analogues (C1, C2 and C7) against HDAC isoforms depicted more selectivity for HDAC2 and HDAC8 (Fig 3B). These acetamides derivatives showed similar binding patterns at Zn^+2^ containing pockets of HDAC2 and 8 (Figures G and H in S1 File), while at the outward surface of binding tunnel, only C1 and C2 showed binding to the PHE (PHE210 of HDAC2 and PHE208 of HDAC8) and TYR (TYR308 of HDAC2 and TYR306 of HDAC8) residues (Figures G and H in S1 File). Similarly, group II (C5 and C6) hydrazides showed binding selectivity against HDAC1, 2 and 3 (Fig 3B). Metal binding regions of C5 and C6 hits formed hydrogen bonds with active site residues of HDAC enzymes, whereas unlike C6, C5 exhibited binding of benzodioxin with HIS178, HIS183, HIS172 of HDAC1, 2 and 3, respectively (Figures I—K in S1 File). Furthermore, predicted bioactivity analysis of these compounds also showed variability where C7 (group I) having dimethoxyphenyl group and C6 (group II) with chlorophenyl substituent exhibited the highest values (12.5 and 8), respectively. (Fig 5).

MD simulations of C8 with HDAC2 and HDAC8 impressively illustrated the conformational readjustments in the corresponding catalytic sites. Generally, certain conformational changes take place in the vicinity of HDAC catalytic sites to accommodate the inhibitor [144,145]. Binding of C8 was mediated by coordination with metal ion and hydrogen bond acceptors such as HIS145, HIS146, HIS183 and TYR308 in HDAC2 and HIS142, HIS143, HIS180 and TYR306 in HDAC8. These residues showed no structural dynamics during the inhibitor binding. Notably, hydrophobic residues namely ILE40-THR43, PHE210 and LEU333 exhibited more fluctuations in HDAC2 bound to C8 hit. Particularly, PHE210 exhibited the highest peak (0.8-2Å) in RMSF plot. This residue is conserved across the class I HDACs. Previously, an active contribution of PHE210 was detected in the binding of NSC746457 [146] and YF479 inhibitor [147] to HDAC2. Moreover, its involvement is also visible in TSA (Trichostatin A)-HDAC2 crystal structure [148]. The consistent trend in residual conformations suggested an active contribution of PHE210 in the induction of inhibitor binding to HDAC2. In HDAC8, more pronounced fluctuations were observed in ASP87, ASP88, SER193-SER199 and GLY320 residues. However, the residues holding the Zn^+2^ ion in the catalytic sites of HDAC2 (ASP181, HIS183 and ASP269) and HDAC8 (HIS142, HIS143, ASP178, HIS180, ASP267 and TYR306) were quite stable.

**Fig 13:**
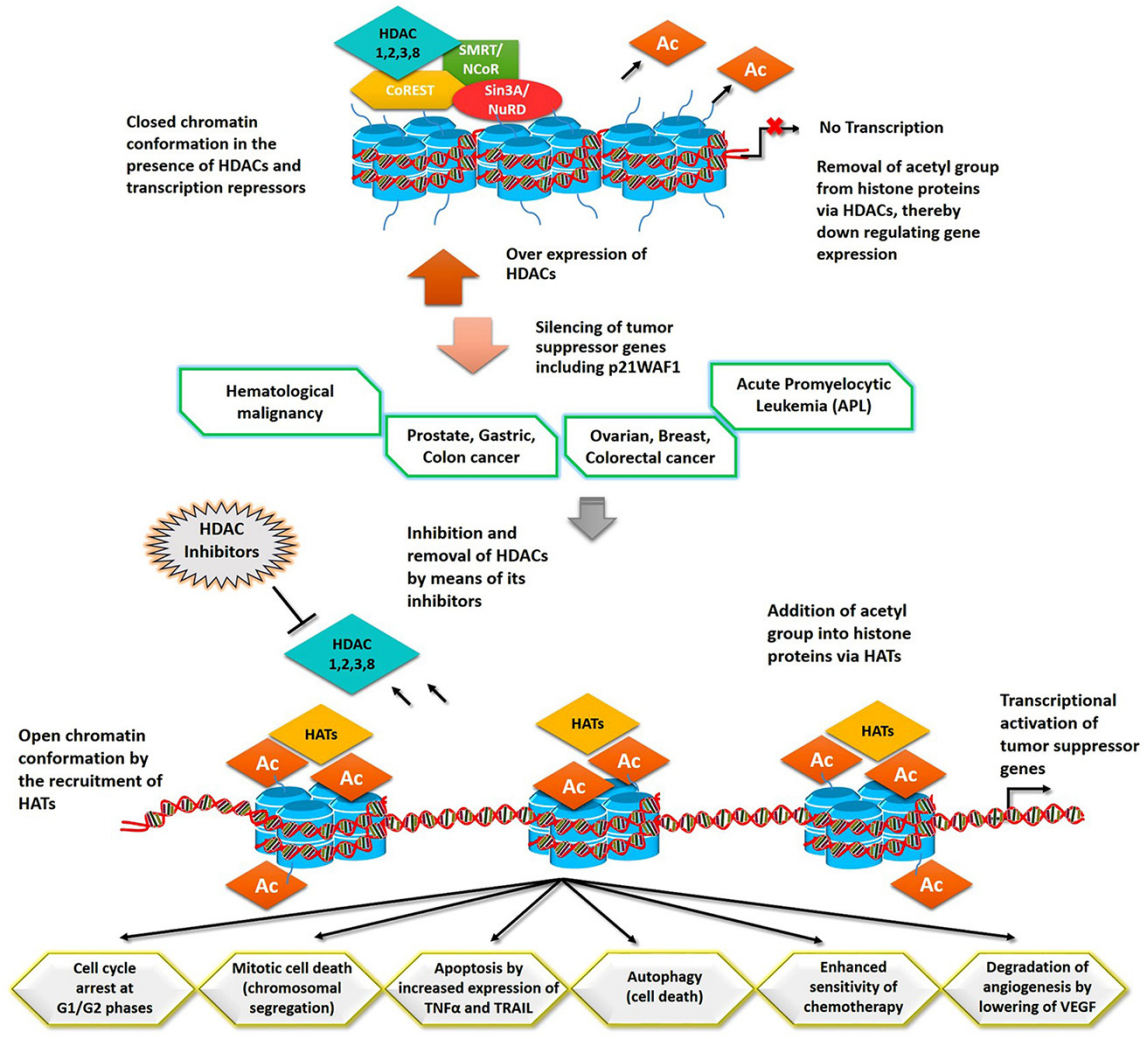
Disease association and inhibition mechanism of class I HDACs. Over expression of HDACs has been perceived in numerous cancers. Binding of drug-like molecules to the catalytic cavity of HDACs eradicates these enzymes from transcription initiation site and stimulates the inclusion of HATs. Acetylation of histones by means of HATs reactivates the transcription of tumor suppressor genes. Expression of these genes controls the abnormal cell growth by cell cycle arrest and mitotic cell death or escalation of apoptosis and autophagy. Tumor suppressor genes enhance the sensitivity of chemotherapy and restrict the process of angiogenesis by decreasing VEGF.

With these structural insights, our nominated drug-like compounds may prove to be operational in combinatorial cancer therapy (Fig 13). Further studies are needed to delineate the inhibitory effects of proposed inhibitors through *in-vitro* and *in-vivo* assays.

## Conclusions

We employed QSAR approach to generate models of 10 chemical compounds (C1-C10) which were derivatives of benzamides, acetamides, carboxamides and hydrazides. Pharmacophore models demonstrated the improved values which stated the higher predictability and reliability of screened hits. The predicted hits were tested for their inhibitory effects through docking and MD simulation assays. C8 and C9 targeted all four members of class I HDACs, whereas other compounds showed member specific interactions. The associated conformational changes in the close proximity of binding regions assisted in the interaction of C8 to HDAC2 and HDAC8. RMSD trends for HDAC2 were much similar in both bound and unbound forms, while HDAC8 bound to C8 attained more stability than apo HDAC8. On the basis of *in-silico* binding analysis, these hits may prove as more potent drugs against class I HDACs than previously known analogs to reverse the disequilibrium of acetylation and deacetylation events.

## Supporting Information

S1 File Supporting information. (PDF)

## References

[1] Legube G, Trouche D. Regulating histone acetyltransferases and deacetylases. EMBO Rep. 2003;4(10): 944–947. 14528264 10.1038/sj.embor.embor941 PMC1326399

[2] Hayes JJ, Tullius TD, Wolffe AP. The structure of DNA in a nucleosome. Proc Natl Acad Sci. 1990;87(19): 7405–7409. 2170977 10.1073/pnas.87.19.7405 PMC54755

[3] Arents G, Moudrianakis EN. Topography of the histone octamer surface: repeating structural motifs utilized in the docking of nucleosomal DNA. Proc Natl Acad Sci. 1993;90(22): 10489–10493. 8248135 10.1073/pnas.90.22.10489 PMC47802

[4] Gregory PD, Wagner K, Hörz W. Histone acetylation and chromatin remodeling. Exp Cell Res. 2001;265(2): 195–202. 11302684 10.1006/excr.2001.5187

[5] Lee DY, Hayes JJ, Pruss D, Wolffe AP. A positive role for histone acetylation in transcription factor access to nucleosomal DNA. Cell. 1993;72(1): 73–84. 8422685 10.1016/0092-8674(93)90051-q

[6] Norton VG, Imai BS, Yau P, Bradbury EM. Histone acetylation reduces nucleosome core particle linking number change. Cell. 1989;57(3): 449–457. 2541913 10.1016/0092-8674(89)90920-3

[7] Vettese-Dadey M, Grant P, Hebbes T, Crane-Robinson C, Allis C, Workman J. Acetylation of histone H4 plays a primary role in enhancing transcription factor binding to nucleosomal DNA in vitro. The EMBO J. 1996;15(10): 2508 8665858 PMC450183

[8] Sidoli S, Cheng L, Jensen ON. Proteomics in chromatin biology and epigenetics: Elucidation of post-translational modifications of histone proteins by mass spectrometry. J Proteomics. 2012;75(12): 3419–3433. 10.1016/j.jprot.2011.12.029 22234360

[9] Berger SL. The complex language of chromatin regulation during transcription. Nature. 2007;447(7143): 407–412. 17522673 10.1038/nature05915

[10] Baradari V, Huether A, Höpfner M, Schuppan D, Scherübl H. Antiproliferative and proapoptotic effects of histone deacetylase inhibitors on gastrointestinal neuroendocrine tumor cells. Endocr-Relat Cancer. 2006;13(4): 1237–1250. 17158768 10.1677/erc.1.01249

[11] Hahn CK, Ross KN, Warrington IM, Mazitschek R, Kanegai CM, Wright RD, et al Expression-based screening identifies the combination of histone deacetylase inhibitors and retinoids for neuroblastoma differentiation. Proc Natl Acad Sci. 2008;105(28): 9751–9756. 10.1073/pnas.0710413105 18607002 PMC2474517

[12] McKinsey TA. Therapeutic potential for HDAC inhibitors in the heart. Annu Rev Pharmacol. 2012;52: 303–319. 10.1146/annurev-pharmtox-010611-134712 21942627

[13] Thomas EA. Focal nature of neurological disorders necessitates isotype-selective histone deacetylase (HDAC) inhibitors. Mol Neurobiol. 2009;40(1): 33–45. 10.1007/s12035-009-8067-y 19396637

[14] De Ruijter A, Van Gennip A, Caron H, Kemp S, van Kuilenburg A. Histone deacetylases (HDACs): characterization of the classical HDAC family. Biochem J. 2003;370: 737–749. 12429021 10.1042/BJ20021321 PMC1223209

[15] Gregoretti I, Lee Y-M, Goodson HV. Molecular evolution of the histone deacetylase family: functional implications of phylogenetic analysis. J Mol Biol. 2004;338(1): 17–31. 15050820 10.1016/j.jmb.2004.02.006

[16] Minucci S, Pelicci PG. Histone deacetylase inhibitors and the promise of epigenetic (and more) treatments for cancer. Nat Rev Cancer. 2006;6(1): 38–51. 16397526 10.1038/nrc1779

[17] Hubbard T, Barker D, Birney E, Cameron G, Chen Y, Clark L, et al The Ensembl genome database project. Nucleic Acids Res. 2002;30(1): 38–41. 11752248 10.1093/nar/30.1.38 PMC99161

[18] Miller KM, Tjeertes JV, Coates J, Legube G, Polo SE, Britton S, et al Human HDAC1 and HDAC2 function in the DNA-damage response to promote DNA nonhomologous end-joining. Nat Struct Mol Biol. 2010;17(99): 1144–1151. 20802485 10.1038/nsmb.1899 PMC3018776

[19] Millard CJ, Watson PJ, Celardo I, Gordiyenko Y, Cowley SM, Robinson CV, et al Class I HDACs share a common mechanism of regulation by inositol phosphates. Mol Cell. 2013;51(1): 57–67. 10.1016/j.molcel.2013.05.020 23791785 PMC3710971

[20] Mercurio C, Minucci S, Pelicci PG. Histone deacetylases and epigenetic therapies of hematological malignancies. Pharmacol Res. 2010;62(1): 18–34. 10.1016/j.phrs.2010.02.010 20219679

[21] Ropero S, Esteller M. The role of histone deacetylases (HDACs) in human cancer. Mol Oncol. 2007;1(1): 19–25. 10.1016/j.molonc.2007.01.001 19383284 PMC5543853

[22] Halkidou K, Gaughan L, Cook S, Leung HY, Neal DE, Robson CN. Upregulation and nuclear recruitment of HDAC1 in hormone refractory prostate cancer. The Prostate. 2004;59(2): 177–189. 15042618 10.1002/pros.20022

[23] Choi JH, Kwon HJ, Yoon BI, Kim JH, Han SU, Joo HJ, et al Expression profile of histone deacetylase 1 in gastric cancer tissues. Cancer Sci. 2001;92(12): 1300–1304. 10.1111/j.1349-7006.2001.tb02153.x PMC5926683 11749695

[24] Zhang Z, Yamashita H, Toyama T, Sugiura H, Ando Y, Mita K, et al Quantitation of HDAC1 mRNA expression in invasive carcinoma of the breast*. Breast Cancer Res Tr. 2005;94(1): 11–16. 10.1007/s10549-005-6001-1 16172792

[25] Wilson AJ, Byun D-S, Popova N, Murray LB, L'Italien K, Sowa Y, et al Histone deacetylase 3 (HDAC3) and other class I HDACs regulate colon cell maturation and p21 expression and are deregulated in human colon cancer. J Biol Chem. 2006;281(19): 13548–13558. 16533812 10.1074/jbc.M510023200

[26] Song J, Noh JH, Lee JH, Eun JW, Ahn YM, Kim SY, et al Increased expression of histone deacetylase 2 is found in human gastric cancer. Apmis. 2005;113(4): 264–268. 15865607 10.1111/j.1600-0463.2005.apm_04.x

[27] Huang B, Laban M, Leung CH, Lee L, Lee C, Salto-Tellez M, et al Inhibition of histone deacetylase 2 increases apoptosis and p21Cip1/WAF1 expression, independent of histone deacetylase 1. Cell Death Differ. 2005;12(4): 395–404. 15665816 10.1038/sj.cdd.4401567

[28] Zhu P, Martin E, Mengwasser J, Schlag P, Janssen K-P, Göttlicher M. Induction of HDAC2 expression upon loss of APC in colorectal tumorigenesis. Cancer cell. 2004;5(5): 455–463. 15144953 10.1016/s1535-6108(04)00114-x

[29] Hayashi A, Horiuchi A, Kikuchi N, Hayashi T, Fuseya C, Suzuki A, et al Type‐specific roles of histone deacetylase (HDAC) overexpression in ovarian carcinoma: HDAC1 enhances cell proliferation and HDAC3 stimulates cell migration with downregulation of E‐cadherin. Int J Cancer. 2010;127(6): 1332–1346. 10.1002/ijc.25151 20049841

[30] Fenrick R, Hiebert SW. Role of histone deacetylases in acute leukemia. J Cell Biochem. 1998;72(S30-31): 194–202. 29345812 10.1002/(SICI)1097-4644(1998)72:30/31+<194::AID-JCB24>3.0.CO;2-H

[31] Mai A, Massa S, Rotili D, Cerbara I, Valente S, Pezzi R, et al Histone deacetylation in epigenetics: an attractive target for anticancer therapy. Med Res Rev. 2005;25(3): 261–309. 15717297 10.1002/med.20024

[32] Di Gennaro E, Bruzzese F, Caraglia M, Abruzzese A, Budillon A. Acetylation of proteins as novel target for antitumor therapy: review article. Amino acids. 2004;26(4): 435–441. 15290351 10.1007/s00726-004-0087-3

[33] Marks PA, Richon VM, Miller T, Kelly WK. Histone deacetylase inhibitors. Adv Cancer Res. 2004;91: 137–168. 15327890 10.1016/S0065-230X(04)91004-4

[34] Dokmanovic M, Marks PA. Prospects: histone deacetylase inhibitors. J Cell Biochem. 2005;96(2): 293–304. 16088937 10.1002/jcb.20532

[35] Bolden JE, Peart MJ, Johnstone RW. Anticancer activities of histone deacetylase inhibitors. Nat Rev Drug Discov. 2006;5(9): 769–784. 16955068 10.1038/nrd2133

[36] Lane AA, Chabner BA. Histone deacetylase inhibitors in cancer therapy. J Clin Oncol. 2009;27(32): 5459–5468. 10.1200/JCO.2009.22.1291 19826124

[37] Monneret C. Histone deacetylase inhibitors. Eur J Med Chem. 2005;40(1): 1–13. 15642405 10.1016/j.ejmech.2004.10.001

[38] Pan L, Lu J, Huang B. HDAC inhibitors: a potential new category of anti-tumor agents. Cell Mol Immunol. 2007;4(5): 337–343. 17976313

[39] Schaefer EW, Loaiza-Bonilla A, Juckett M, DiPersio JF, Roy V, Slack J, et al A phase 2 study of vorinostat in acute myeloid leukemia. haematologica. 2009;94(10): 1375–1382. 10.3324/haematol.2009.009217 19794082 PMC2754953

[40] Molife L, Attard G, Fong P, Karavasilis V, Reid A, Patterson S, et al Phase II, two-stage, single-arm trial of the histone deacetylase inhibitor (HDACi) romidepsin in metastatic castration-resistant prostate cancer (CRPC). ANN ONCOL. 2010;21(1): 109–113. 10.1093/annonc/mdp270 19608618

[41] Mackay HJ, Hirte H, Colgan T, Covens A, MacAlpine K, Grenci P, et al Phase II trial of the histone deacetylase inhibitor belinostat in women with platinum resistant epithelial ovarian cancer and micropapillary (LMP) ovarian tumours. Eur J Cancer. 2010;46(9): 1573–1579. 10.1016/j.ejca.2010.02.047 20304628 PMC3244274

[42] Wagner JM, Hackanson B, Lübbert M, Jung M. Histone deacetylase (HDAC) inhibitors in recent clinical trials for cancer therapy. Clin Epigenetics. 2010;1(3–4): 117–136. 21258646 10.1007/s13148-010-0012-4 PMC3020651

[43] Kim H-J, Bae S-C. Histone deacetylase inhibitors: molecular mechanisms of action and clinical trials as anti-cancer drugs. Am J Transl Res. 2011;3(2): 166 21416059 PMC3056563

[44] Younes A, Sureda A, Ben-Yehuda D, Zinzani PL, Ong T-C, Prince HM, et al Panobinostat in patients with relapsed/refractory Hodgkin's lymphoma after autologous stem-cell transplantation: results of a phase II study. J Clin Oncol. 2012;30(18): 2197–2203. 10.1200/JCO.2011.38.1350 22547596

[45] KIM YB, KI SW, YOSNIDA M, HORINOUCHI S. Mechanism of cell cycle arrest caused by histone deacetylase inhibitors in human carcinoma cells. J Antibiot. 2000;53(10): 1191–1200. 11132966 10.7164/antibiotics.53.1191

[46] Geng L, Cuneo KC, Fu A, Tu T, Atadja PW, Hallahan DE. Histone Deacetylase (HDAC) Inhibitor LBH589 Increases Duration of γ-H2AX Foci and Confines HDAC4 to the Cytoplasm in Irradiated Non–Small Cell Lung Cancer. Cancer Res. 2006;66(23): 11298–11304. 17145876 10.1158/0008-5472.CAN-06-0049

[47] Wang D-F, Helquist P, Wiech NL, Wiest O. Toward selective histone deacetylase inhibitor design: homology modeling, docking studies, and molecular dynamics simulations of human class I histone deacetylases. J Med Chem. 2005;48(22): 6936–6947. 16250652 10.1021/jm0505011

[48] Vadivelan S, Sinha B, Rambabu G, Boppana K, Jagarlapudi SA. Pharmacophore modeling and virtual screening studies to design some potential histone deacetylase inhibitors as new leads. J Mol Graphics Modell. 2008;26(6): 935–946. 10.1016/j.jmgm.2007.07.002 17707666

[49] Tang H, Wang XS, Huang X-P, Roth BL, Butler KV, Kozikowski AP, et al Novel inhibitors of human histone deacetylase (HDAC) identified by QSAR modeling of known inhibitors, virtual screening, and experimental validation. J Chem Inf Model. 2009;49(2): 461–476. 10.1021/ci800366f 19182860

[50] Melagraki G, Afantitis A, Sarimveis H, Koutentis PA, Kollias G, Igglessi-Markopoulou O. Predictive QSAR workflow for the in silico identification and screening of novel HDAC inhibitors. Mol Divers. 2009;13(3): 301–311. 10.1007/s11030-009-9115-2 19205914

[51] Nair SB, Teli MK, Pradeep H, Rajanikant G. Computational identification of novel histone deacetylase inhibitors by docking based QSAR. Comput Biol Med. 2012;42(6): 697–705. 10.1016/j.compbiomed.2012.04.001 22521374

[52] Park H, Kim S, Kim YE, Lim SJ. A Structure‐Based Virtual Screening Approach toward the Discovery of Histone Deacetylase Inhibitors: Identification of Promising Zinc‐Chelating Groups. ChemMedChem. 2010;5(4): 591–597. 10.1002/cmdc.200900500 20157916

[53] Kalyaanamoorthy S, Chen Y-PP. Energy based pharmacophore mapping of HDAC inhibitors against class I HDAC enzymes. BBA-Proteins Proteom. 2013;1834(1): 317–328. 10.1016/j.bbapap.2012.08.009 23457710

[54] Berman HM, Westbrook J, Feng Z, Gilliland G, Bhat T, Weissig H, et al The protein data bank. Nucleic Acids Res. 2000;28(1): 235–242. 10592235 10.1093/nar/28.1.235 PMC102472

[55] Pettersen EF, Goddard TD, Huang CC, Couch GS, Greenblatt DM, Meng EC, et al UCSF Chimera—a visualization system for exploratory research and analysis. J Comput Chem. 2004;25(13): 1605–1612. 15264254 10.1002/jcc.20084

[56] Laskowski RA. PDBsum: summaries and analyses of PDB structures. Nucleic Acids Res. 2001;29(1): 221–222. 11125097 10.1093/nar/29.1.221 PMC29784

[57] Remiszewski SW, Sambucetti LC, Atadja P, Bair KW, Cornell WD, Green MA, et al Inhibitors of human histone deacetylase: synthesis and enzyme and cellular activity of straight chain hydroxamates. J Med Chem. 2002;45(4): 753–757. 11831887 10.1021/jm015568c

[58] Kim D-K, Lee JY, Kim J-S, Ryu J-H, Choi J-Y, Lee JW, et al Synthesis and biological evaluation of 3-(4-substituted-phenyl)-N-hydroxy-2-propenamides, a new class of histone deacetylase inhibitors. J Med Chem. 2003;46(26): 5745–5751. 14667227 10.1021/jm030377q

[59] Bouchain G, Leit S, Frechette S, Khalil EA, Lavoie R, Moradei O, et al Development of potential antitumor agents. Synthesis and biological evaluation of a new set of sulfonamide derivatives as histone deacetylase inhibitors. J Med Chem. 2003;46(5): 820–830. 12593661 10.1021/jm020377a

[60] Gurvich N, Tsygankova OM, Meinkoth JL, Klein PS. Histone deacetylase is a target of valproic acid-mediated cellular differentiation. Cancer Res. 2004;64(3): 1079–1086. 14871841 10.1158/0008-5472.can-03-0799

[61] Moradei OM, Mallais TC, Frechette S, Paquin I, Tessier PE, Leit SM, et al Novel aminophenyl benzamide-type histone deacetylase inhibitors with enhanced potency and selectivity. J Med Chem. 2007;50(23): 5543–5546. 17941625 10.1021/jm701079h

[62] Witter DJ, Harrington P, Wilson KJ, Chenard M, Fleming JC, Haines B, et al Optimization of biaryl selective HDAC1&2 inhibitors (SHI-1: 2). Bioorg Med Chem Lett. 2008;18(2): 726–731. 18060775 10.1016/j.bmcl.2007.11.047

[63] Zhou N, Moradei O, Raeppel S, Leit S, Frechette S, Gaudette F, et al Discovery of N-(2-aminophenyl)-4-[(4-pyridin-3-ylpyrimidin-2-ylamino) methyl] benzamide (MGCD0103), an orally active histone deacetylase inhibitor. J Med Chem. 2008;51(14): 4072–4075. 10.1021/jm800251w 18570366

[64] Jones P, Altamura S, De Francesco R, Gallinari P, Lahm A, Neddermann P, et al Probing the elusive catalytic activity of vertebrate class IIa histone deacetylases. Bioorg Med Chem Lett. 2008;18(6): 1814–1819. 10.1016/j.bmcl.2008.02.025 18308563

[65] Kinzel O, Llauger-Bufi L, Pescatore G, Rowley M, Schultz-Fademrecht C, Monteagudo E, et al Discovery of a potent class I selective ketone histone deacetylase inhibitor with antitumor activity in vivo and optimized pharmacokinetic properties. J Med Chem. 2009;52(11): 3453–3456. 10.1021/jm9004303 19441846

[66] Olsen CA, Ghadiri MR. Discovery of potent and selective histone deacetylase inhibitors via focused combinatorial libraries of cyclic α3β-tetrapeptides. J Med Chem. 2009;52(23): 7836–7846. 10.1021/jm900850t 19705846 PMC2788660

[67] Hutt DM, Herman D, Rodrigues AP, Noel S, Pilewski JM, Matteson J, et al Reduced histone deacetylase 7 activity restores function to misfolded CFTR in cystic fibrosis. Nat Chem Biol. 2010;6(1): 25–33. 10.1038/nchembio.275 19966789 PMC2901172

[68] Auzzas L, Larsson A, Matera R, Baraldi A, Deschenes-Simard Bt, Giannini G, et al Non-natural macrocyclic inhibitors of histone deacetylases: design, synthesis, and activity. J Med Chem. 2010;53(23): 8387–8399. 10.1021/jm101092u 21073160

[69] Vaidya AS, Karumudi B, Mendonca E, Madriaga A, Abdelkarim H, van Breemen RB, et al Design, synthesis, modeling, biological evaluation and photoaffinity labeling studies of novel series of photoreactive benzamide probes for histone deacetylase 2. Bioorg Med Chem Lett. 2012;22(15): 5025–5030. 10.1016/j.bmcl.2012.06.017 22771007 PMC3401313

[70] Hirata Y, Hirata M, Kawaratani Y, Shibano M, Taniguchi M, Yasuda M, et al Anti-tumor activity of new orally bioavailable 2-amino-5-(thiophen-2-yl) benzamide-series histone deacetylase inhibitors, possessing an aqueous soluble functional group as a surface recognition domain. Bioorg Med Chem Lett. 2012;22(5): 1926–1930. 10.1016/j.bmcl.2012.01.053 22321215

[71] Olson DE, Wagner FF, Kaya T, Gale JP, Aidoud N, Davoine EL, et al Discovery of the first histone deacetylase 6/8 dual inhibitors. J Med Chem. 2013;56(11): 4816–4820. 10.1021/jm400390r 23672185

[72] Feng T, Wang H, Su H, Lu H, Yu L, Zhang X, et al Novel N-hydroxyfurylacrylamide-based histone deacetylase (HDAC) inhibitors with branched CAP group (Part 2). Bioorgan Med Chem. 2013;21(17): 5339–5354. 10.1016/j.bmc.2013.06.009 23820574

[73] Kalin JH, Bergman JA. Development and therapeutic implications of selective histone deacetylase 6 inhibitors. J Med Chem. 2013;56(16): 6297–6313. 10.1021/jm4001659 23627282

[74] Gaulton A, Bellis LJ, Bento AP, Chambers J, Davies M, Hersey A, et al ChEMBL: a large-scale bioactivity database for drug discovery. Nucleic Acids Res. 2012;40(D1): D1100–D1107. 21948594 10.1093/nar/gkr777 PMC3245175

[75] Swain M. Chemicalize. org. J Chem Inf Model. 2012;52(2): 613–615.

[76] Cheminformatics M. Molinspiration. 2013.

[77] Trott O, Olson AJ. AutoDock Vina: improving the speed and accuracy of docking with a new scoring function, efficient optimization, and multithreading. J Comput Chem. 2010;31(2): 455–461. 10.1002/jcc.21334 19499576 PMC3041641

[78] Summers JB, Kim KH, Mazdiyasni H, Holms JH, Ratajczyk JD, Stewart AO, et al Hydroxamic acid inhibitors of 5-lipoxygenase: quantitative structure-activity relationships. J Med Chem. 1990;33(3): 992–998. 2308149 10.1021/jm00165a017

[79] Wu TY, Hassig C, Wu Y, Ding S, Schultz PG. Design, synthesis, and activity of HDAC inhibitors with a N-formyl hydroxylamine head group. Bioorg Med Chem Lett. 2004;14(2): 449–453. 14698179 10.1016/j.bmcl.2003.10.055

[80] Jones P, Altamura S, Chakravarty PK, Cecchetti O, De Francesco R, Gallinari P, et al A series of novel, potent, and selective histone deacetylase inhibitors. Bioorg Med Chem Lett. 2006;16(23): 5948–5952. 16987657 10.1016/j.bmcl.2006.09.002

[81] Mahboobi S, Sellmer A, Höcher H, Garhammer C, Pongratz H, Maier T, et al 2-Aroylindoles and 2-aroylbenzofurans with N-hydroxyacrylamide substructures as a novel series of rationally designed histone deacetylase inhibitors. J Med Chem. 2007;50(18): 4405–4418. 17691763 10.1021/jm0703136

[82] Kozikowski AP, Chen Y, Gaysin A, Chen B, D'Annibale MA, Suto CM, et al Functional differences in epigenetic modulators superiority of mercaptoacetamide-based histone deacetylase inhibitors relative to hydroxamates in cortical neuron neuroprotection studies. J Med Chem. 2007;50(13): 3054–3061. 17539623 10.1021/jm070178x

[83] Methot JL, Chakravarty PK, Chenard M, Close J, Cruz JC, Dahlberg WK, et al Exploration of the internal cavity of histone deacetylase (HDAC) with selective HDAC1/HDAC2 inhibitors (SHI-1: 2). Bioorg Med Chem Lett. 2008;18(3): 973–978. 10.1016/j.bmcl.2007.12.031 18182289

[84] Kozikowski AP, Tapadar S, Luchini DN, Kim KH, Billadeau DD. Use of the nitrile oxide cycloaddition (NOC) reaction for molecular probe generation: a new class of enzyme selective histone deacetylase inhibitors (HDACIs) showing picomolar activity at HDAC6. J Med Chem. 2008;51(15): 4370–4373. 10.1021/jm8002894 18642892 PMC3913184

[85] Chen Y, Lopez-Sanchez M, Savoy DN, Billadeau DD, Dow GS, Kozikowski AP. A series of potent and selective, triazolylphenyl-based histone deacetylases inhibitors with activity against pancreatic cancer cells and Plasmodium falciparum. J Med Chem. 2008;51(12): 3437–3448. 10.1021/jm701606b 18494463

[86] Tapadar S, He R, Luchini DN, Billadeau DD, Kozikowski AP. Isoxazole moiety in the linker region of HDAC inhibitors adjacent to the Zn-chelating group: effects on HDAC biology and antiproliferative activity. Bioorg Med Chem Lett. 2009;19(11): 3023–3026. 10.1016/j.bmcl.2009.04.058 19419863 PMC2705901

[87] Dallavalle S, Cincinelli R, Nannei R, Merlini L, Morini G, Penco S, et al Design, synthesis, and evaluation of biphenyl-4-yl-acrylohydroxamic acid derivatives as histone deacetylase (HDAC) inhibitors. Eur J Med Chem. 2009;44(5): 1900–1912. 10.1016/j.ejmech.2008.11.005 19084294

[88] Gupta PK, Reid RC, Liu L, Lucke AJ, Broomfield SA, Andrews MR, et al Inhibitors selective for HDAC6 in enzymes and cells. Bioorg Med Chem Lett. 2010;20(23): 7067–7070. 10.1016/j.bmcl.2010.09.100 20947351

[89] He R, Chen Y, Chen Y, Ougolkov AV, Zhang J-S, Savoy DN, et al Synthesis and biological evaluation of triazol-4-ylphenyl-bearing histone deacetylase inhibitors as anticancer agents. J Med Chem. 2010;53(3): 1347–1356. 10.1021/jm901667k 20055418 PMC2919064

[90] Cho YS, Whitehead L, Li J, Chen CH-T, Jiang L, Vögtle M, et al Conformational refinement of hydroxamate-based histone deacetylase inhibitors and exploration of 3-piperidin-3-ylindole analogues of dacinostat (LAQ824). J Med Chem. 2010;53(7): 2952–2963. 10.1021/jm100007m 20205394

[91] Oger F, Lecorgne A, Sala E, Nardese V, Demay F, Chevance S, et al Biological and biophysical properties of the histone deacetylase inhibitor suberoylanilide hydroxamic acid are affected by the presence of short alkyl groups on the phenyl ring. J Med Chem. 2010;53(5): 1937–1950. 10.1021/jm901561u 20143840

[92] Bressi JC, Jennings AJ, Skene R, Wu Y, Melkus R, De Jong R, et al Exploration of the HDAC2 foot pocket: Synthesis and SAR of substituted N-(2-aminophenyl) benzamides. Bioorg Med Chem Lett. 2010;20(10): 3142–3145. 10.1016/j.bmcl.2010.03.091 20392638

[93] Terracciano S, Di Micco S, Bifulco G, Gallinari P, Riccio R, Bruno I. Synthesis and biological activity of cyclotetrapeptide analogues of the natural HDAC inhibitor FR235222. Bioorgan Med Chem. 2010;18(9): 3252–3260. 10.1016/j.bmc.2010.03.022 20381359

[94] Mahboobi S, Sellmer A, Winkler M, Eichhorn E, Pongratz H, Ciossek T, et al Novel chimeric histone deacetylase inhibitors: a series of lapatinib hybrides as potent inhibitors of epidermal growth factor receptor (EGFR), human epidermal growth factor receptor 2 (HER2), and histone deacetylase activity. J Med Chem. 2010;53(24): 8546–8555. 10.1021/jm100665z 21080629

[95] Kemp MM, Wang Q, Fuller JH, West N, Martinez NM, Morse EM, et al A novel HDAC inhibitor with a hydroxy-pyrimidine scaffold. Bioorg Med Chem Lett. 2011;21(14): 4164–4169. 10.1016/j.bmcl.2011.05.098 21696956 PMC3248787

[96] Botta CB, Cabri W, Cini E, De Cesare L, Fattorusso C, Giannini G, et al Oxime amides as a novel zinc binding group in histone deacetylase inhibitors: synthesis, biological activity, and computational evaluation. J Med Chem. 2011;54(7): 2165–2182. 10.1021/jm101373a 21417297

[97] Neelarapu R, Holzle DL, Velaparthi S, Bai H, Brunsteiner M, Blond SY, et al Design, synthesis, docking, and biological evaluation of novel diazide-containing isoxazole-and pyrazole-based histone deacetylase probes. J Med Chem. 2011;54(13): 4350–4364. 10.1021/jm2001025 21548582 PMC3466118

[98] Huang D, Li X, Wei Y, Xiu Z. A novel series of l-2-benzyloxycarbonylamino-8-(2-pyridyl)-disulfidyloctanoic acid derivatives as histone deacetylase inhibitors: design, synthesis and molecular modeling study. Eur J Med Chem. 2012;52: 111–122. 10.1016/j.ejmech.2012.03.009 22465091

[99] Lai M-J, Huang H-L, Pan S-L, Liu Y-M, Peng C-Y, Lee H-Y, et al Synthesis and biological evaluation of 1-arylsulfonyl-5-(N-hydroxyacrylamide) indoles as potent histone deacetylase inhibitors with antitumor activity in vivo. J Med Chem. 2012;55(8): 3777–3791. 10.1021/jm300197a 22439863 PMC4201585

[100] Wagner FF, Olson DE, Gale JP, Kaya T, Weïwer M, Aidoud N, et al Potent and selective inhibition of histone deacetylase 6 (HDAC6) does not require a surface-binding motif. J Med Chem. 2013;56(4): 1772–1776. 10.1021/jm301355j 23368884

[101] Miller TA, Witter DJ, Belvedere S. Histone deacetylase inhibitors. J Med Chem. 2003;46(24): 5097–5116. 14613312 10.1021/jm0303094

[102] Chen J-B, Chern T-R, Wei T-T, Chen C-C, Lin J-H, Fang J-M. Design and synthesis of dual-action inhibitors targeting histone deacetylases and 3-hydroxy-3-methylglutaryl coenzyme A reductase for cancer treatment. J Med Chem. 2013;56(9): 3645–3655. 10.1021/jm400179b 23570542

[103] Marson CM, Matthews CJ, Yiannaki E, Atkinson SJ, Soden PE, Shukla L, et al Discovery of potent, isoform-selective inhibitors of histone deacetylase containing chiral heterocyclic capping groups and a N-(2-aminophenyl) benzamide binding unit. J Med Chem. 2013;56(15): 6156–6174. 10.1021/jm400634n 23829483

[104] Wang T, Sepulveda M, Gonzales P, Gately S. Identification of novel HDAC inhibitors through cell based screening and their evaluation as potential anticancer agents. Bioorg Med Chem Lett. 2013;23(17): 4790–4793. 10.1016/j.bmcl.2013.07.001 23906422

[105] Yu C-W, Chang P-T, Hsin L-W, Chern J-W. Quinazolin-4-one derivatives as selective histone deacetylase-6 inhibitors for the treatment of Alzheimer’s disease. J Med Chem. 2013;56(17): 6775–6791. 10.1021/jm400564j 23905680

[106] Blackburn C, Barrett C, Chin J, Garcia K, Gigstad K, Gould A, et al Potent histone deacetylase inhibitors derived from 4-(aminomethyl)-N-hydroxybenzamide with high selectivity for the HDAC6 isoform. J Med Chem. 2013;56(18): 7201–7211. 10.1021/jm400385r 23964961

[107] Guerra-Bubb JM, Bowers AA, Smith WB, Paranal R, Estiu G, Wiest O, et al Synthesis and HDAC inhibitory activity of isosteric thiazoline-oxazole largazole analogs. Bioorg Med Chem Lett. 2013;23(21): 6025–6028. 10.1016/j.bmcl.2013.06.012 24035339 PMC3859309

[108] Zhang X, Bao B, Yu X, Tong L, Luo Y, Huang Q, et al The discovery and optimization of novel dual inhibitors of topoisomerase II and histone deacetylase. Bioorgan Med Chem. 2013;21(22): 6981–6995. 10.1016/j.bmc.2013.09.023 24095018

[109] Taddei M, Cini E, Giannotti L, Giannini G, Battistuzzi G, Vignola D, et al Lactam based 7-amino suberoylamide hydroxamic acids as potent HDAC inhibitors. Bioorg Med Chem Lett. 2014;24(1): 61–64. 10.1016/j.bmcl.2013.11.072 24345446

[110] ACD/ChemSketch Freeware, Advanced Chemistry Development, Inc., Toronto, ON, Canada, www.acdlabs.com, 2014.

[111] Wolber G, Langer T. LigandScout: 3-D pharmacophores derived from protein-bound ligands and their use as virtual screening filters. J Chem Inf Model. 2005;45(1): 160–169. 15667141 10.1021/ci049885e

[112] Shahlaei M. Descriptor selection methods in quantitative structure–activity relationship studies: a review study. Chem Rev. 2013;113(10): 8093–8103. 10.1021/cr3004339 23822589

[113] Verma J, Khedkar VM, Coutinho EC. 3D-QSAR in drug design-a review. Curr Top Med Chem. 2010;10(1): 95–115. 19929826 10.2174/156802610790232260

[114] IBM SPSS statistics for windows (2012). Corporation, I. B. M, 22 edn.,

[115] Aurora Fine Chemicals Ltd–Europe. http://www.aurorafinechemicals.com.

[116] Princeton Biomolecular Research, Inc. http://www.princetonbio.com.

[117] Loeffler HH, Winn M. Large biomolecular simulation on hpc platforms III. AMBER, CHARMM, GROMACS, LAMMPS and NAMD. Technical report, STFC Daresbury Laboratory, Warrington WA4 4AD, UK, 2012.

[118] Bibi N, Parveen Z, Rashid S. Identification of potential Plk1 targets in a cell-cycle specific proteome through structural dynamics of kinase and Polo box-mediated interactions. PloS one. 2013;8(8): e70843 10.1371/journal.pone.0070843 23967120 PMC3744538

[119] Kausar S, Asif M, Bibi N, Rashid S. Correction: Comparative Molecular Docking Analysis of Cytoplasmic Dynein Light Chain DYNLL1 with Pilin to Explore the Molecular Mechanism of Pathogenesis Caused by Pseudomonas aeruginosa PAO. PloS one. 2013;8(11). 10.1371/journal.pone.0076730 PMC3789673 24098557

[120] Discovery Studio Modeling Environment (2013). Accelrys Software Inc, 4.0 edn., San Diego

[121] Cheng F, Li W, Zhou Y, Shen J, Wu Z, Liu G, et al admetSAR: a comprehensive source and free tool for assessment of chemical ADMET properties. J Chem Inf Model. 2012;52(11): 3099–3105. 10.1021/ci300367a 23092397

[122] Sander T. OSIRIS property explorer Allschwil: Actelion Pharmaceuticals Ltd 2001

[123] Boda K, Seidel T, Gasteiger J. Structure and reaction based evaluation of synthetic accessibility. J Comput Aid Mol Des. 2007;21(6): 311–325. 10.1007/s10822-006-9099-2 17294248

[124] Seidel T, Ibis G, Bendix F, Wolber G. Strategies for 3D pharmacophore-based virtual screening. Drug Discov Today: Technol. 2011;7(4): e221–e228. 10.1016/j.ddtec.2010.11.004 24103798

[125] Tropsha A. Best practices for QSAR model development, validation, and exploitation. Mol Inform. 2010;29(6‐7): 476–488. 27463326 10.1002/minf.201000061

[126] Andrews P, Craik D, Martin J. Functional group contributions to drug-receptor interactions. J Med Chem. 1984;27(12): 1648–1657. 6094812 10.1021/jm00378a021

[127] Reynolds CH, Bembenek SD, Tounge BA. The role of molecular size in ligand efficiency. Bioorg Med Chem Lett. 2007;17(15): 4258–4261. 17532632 10.1016/j.bmcl.2007.05.038

[128] Reynolds CH, Tounge BA, Bembenek SD. Ligand binding efficiency: trends, physical basis, and implications. J Med Chem. 2008;51(8): 2432–2438. 10.1021/jm701255b 18380424

[129] Leeson PD, Springthorpe B. The influence of drug-like concepts on decision-making in medicinal chemistry. Nat Rev Drug Discov. 2007;6(11): 881–890. 17971784 10.1038/nrd2445

[130] Ryckmans T, Edwards MP, Horne VA, Correia AM, Owen DR, Thompson LR, et al Rapid assessment of a novel series of selective CB 2 agonists using parallel synthesis protocols: a lipophilic efficiency (LipE) analysis. Bioorg Med Chem Lett. 2009;19(15): 4406–4409. 10.1016/j.bmcl.2009.05.062 19500981

[131] Keserü GM, Makara GM. The influence of lead discovery strategies on the properties of drug candidates. Nat Rev Drug Discov. 2009;8(3): 203–212. 10.1038/nrd2796 19247303

[132] Mortenson PN, Murray CW. Assessing the lipophilicity of fragments and early hits. J Comput Aid Mol Des. 2011;25(7): 663–667. 10.1007/s10822-011-9435-z 21614595

[133] Hopkins AL, Keserü GM, Leeson PD, Rees DC, Reynolds CH. The role of ligand efficiency metrics in drug discovery. Nat Rev Drug Discov. 2014;13(2): 105–121. 10.1038/nrd4163 24481311

[134] Bio-Loom for Windows version 1.5. BioByte Corporation, CA, USA

[135] Kuntz I, Chen K, Sharp K, Kollman P. The maximal affinity of ligands. Proc Natl Acad Sci. 1999;96(18): 9997–10002. 10468550 10.1073/pnas.96.18.9997 PMC17830

[136] Giannini G, Vesci L, Battistuzzi G, Vignola D, Milazzo FM, Guglielmi MB, et al ST7612AA1, a Thioacetate-ω (γ-lactam carboxamide) Derivative Selected from a Novel Generation of Oral HDAC Inhibitors. J Med Chem. 2014;57(20): 8358–8377. 10.1021/jm5008209 25233084

[137] Attenni B, Ontoria JM, Cruz JC, Rowley M, Schultz-Fademrecht C, Steinkühler C, et al Histone deacetylase inhibitors with a primary amide zinc binding group display antitumor activity in xenograft model. Bioorg Med Chem Lett. 2009;19(11): 3081–3084. 10.1016/j.bmcl.2009.04.011 19410459

[138] Hranjec M, Sović I, Ratkaj I, Pavlović G, Ilić N, Valjalo L, et al Antiproliferative potency of novel benzofuran-2-carboxamides on tumour cell lines: cell death mechanisms and determination of crystal structure. Eur J Med Chem. 2013;59: 111–119. 10.1016/j.ejmech.2012.11.009 23220640

[139] Baraldi PG, Romagnoli R, Beria I, Cozzi P, Geroni C, Mongelli N, et al Synthesis and antitumor activity of new benzoheterocyclic derivatives of distamycin A. J Med Chem. 2000;43(14): 2675–2684. 10893305 10.1021/jm9911229

[140] Baraldi PG, Romagnoli R, Bianchi N, Gambari R. Benzoyl nitrogen mustard derivatives of benzoheterocyclic analogues of netropsin: synthesis and biological activity. Bioorgan Med Chem. 2003;11(11): 2381–2388. 10.1016/s0968-0896(03)00144-5 12735983

[141] Wang Y, Li L, Ye W, Tian Z, Jiang W, Wang H, et al CC-1065 analogues bearing different DNA-binding subunits: synthesis, antitumor activity, and preliminary toxicity study. J Med Chem. 2003;46(4): 634–637. 12570384 10.1021/jm0203433

[142] Saku O, Saki M, Kurokawa M, Ikeda K, Takizawa T, Uesaka N. Synthetic studies on selective adenosine A 2A receptor antagonists: Synthesis and structure–activity relationships of novel benzofuran derivatives. Bioorg Med Chem Lett. 2010;20(3): 1090–1093. 10.1016/j.bmcl.2009.12.028 20034788

[143] Mai A, Massa S, Pezzi R, Simeoni S, Rotili D, Nebbioso A, et al Class II (IIa)-selective histone deacetylase inhibitors. 1. Synthesis and biological evaluation of novel (aryloxopropenyl) pyrrolyl hydroxyamides. J Med Chem. 2005;48(9): 3344–3353. 15857140 10.1021/jm049002a

[144] Park H, Lee S. Homology modeling, force field design, and free energy simulation studies to optimize the activities of histone deacetylase inhibitors. J Comput Aid Mol Des. 2004;18(6): 375–388. 10.1007/s10822-004-2283-3 15662999

[145] Yan C, Xiu Z, Li X, Li S, Hao C, Teng H. Comparative molecular dynamics simulations of histone deacetylase‐like protein: Binding modes and free energy analysis to hydroxamic acid inhibitors. Proteins: Struct, Funct, Bioinf. 2008;73(1): 134–149. 10.1002/prot.22047 18398905

[146] Hou J, Feng C, Li Z, Fang Q, Wang H, Gu G, et al Structure-based optimization of click-based histone deacetylase inhibitors. Eur J Med Chem. 2011;46(8): 3190–3200. 10.1016/j.ejmech.2011.04.027 21621883

[147] Zhang T, Chen Y, Li J, Yang F, Wu H, Dai F, et al Antitumor action of a novel histone deacetylase inhibitor, YF479, in breast cancer. Neoplasia. 2014;16(8): 665–677. 10.1016/j.neo.2014.07.009 25220594 PMC4234873

[148] Cronin CN, Hilgers MT, Knuth MW, Navre ME, Sang BC, Skene RJ, et al. Crystallization of histone deacetylase 2. Google Patents; 2009.

