# Correction: Exploration of Novel Inhibitors for Class I Histone Deacetylase Isoforms by QSAR Modeling and Molecular Dynamics Simulation Assays

**DOI:** 10.1371/journal.pone.0143155

**Published:** 2015-11-12

**Authors:** Zainab Noor, Noreen Afzal, Sajid Rashid

There are formatting errors in Table 1. Please see the corrected Table 1 below.

**Table 1:** Classification and biological roles of HDACs.

Classes	Cofactors	HDACs	Cellular Locations	Biological Processes	References
Class I	Zn^+2^ dependent	1, 2, 3, 8	Nucleus, Cytoplasm, Transcriptional repressor complex, Spindle microtubule, Replication fork	Cell cycle regulation, Cell differentiation, DNA damage response, Epidermis development, Regulating cardiac myocyte proliferation on the course of cardiac development	[16–18]
Class II	Zn^+2^ dependent	4, 5, 6, 7, 9, 10	Cytoplasm, Nucleus, Neuromuscular junction, Golgi apparatus, Cytosol caveola	Regulation of transcription and cell differentiation, Regulation of cardiac muscle contraction, Inflammatory response, Nervous system development, Heart development, Protein polyubiquitination, Response to toxic and organic substances, Macroautophagy, Vasculogenesis	[1, 17]
Class III	NAD^+^ dependent	Sirtuins SIRT1-SIRT7	Nucleus, Cytoplasm	Histone deacetylation, Regulation of phosphorylation, Regulation of double-strand break repair via homologous recombination, DNA repair mechanism	[16]
Class IV	Zn^+2^ dependent	11	Nucleus	Transcription, DNA-dependent chromatin modification, Histone Deacetylation	[16]
