# Supplementary material for: Exploration of Novel Inhibitors for Class I Histone Deacetylase Isoforms by QSAR Modeling and Molecular Dynamics Simulation Assays
Source: PLoS One. 2015 Oct 2;10(10):e0139588. doi: 10.1371/journal.pone.0139588 (PMC4592208; doi:10.1371/journal.pone.0139588)
Supplement: S1 File — (PDF) [file pone.0139588.s001.pdf]

Exploration of Novel Inhibitors for Class I Histone Deacetylase Isoforms by QSAR Modeling and Molecular Dynamics Simulation Assays

Zainab Noor, Noreen Afzal, Sajid Rashid*

Quaid-i-Azam University, Islamabad

***Email:** sajidrwp@yahoo.co.uk

Supplementary Material

Table A PDB ids of class 1 HDAC target proteins

Protein Name	PDB id	Resolutions	Date
**HDAC1**	4BKX	3.00 Å	2013
**HDAC2**	4LXZ	1.85 Å	2013
**HDAC3**	4A69	2.06 Å	2012
**HDAC8**	1T64	1.90 Å	2004

Table B IC_{50} values of 16 known class I HDAC inhibitors

Sr. No.	Compounds	IC_{50} values (nM)			
		**HDAC1**	**HDAC2**	**HDAC3**	**HDAC8**
**1.**	MS-275	120	250	120	>1000
**2.**	CI-994	900	900	1200	>2000
**3.**	LAQ-824	3.23	15.70	10.50	3.84
**4.**	LBH-589	6.8	<300	<300	<300
**5.**	Belinostat	18	-	46	-
**6.**	SK-7041	980	980	-	-
**7.**	SAHA	30	10	17	110
**8.**	Trichostatin A	5	18	2	25
**9.**	Oxamflatin	100	233.1	233.1	233.1
**10.**	Pyroxamide	200	200	200	200
**11.**	Valproic Acid	39000	62000	161000	103000
**12.**	Scriptaid	200	700	100	300
**13.**	Depsipeptide	36	1	-	-
**14.**	Phenyl Butyrate	65000	-	260000	9300
**15.**	Tubacin	1400	6270	1270	1270
**16.**	Mocetinostat	150	290	610	>1000

Table C IC50 (nM) values and SMILE codes for test set compounds. Test set compounds were labelled with the unique identifiers i.e. compound number- ChEMBL ID

ID	IC_{50}	SMILE code	ID	IC_{50}	SMILE code
**1-16300**	**9-20000**	ONC(=O)C1=CC=CC=C1	**19-487107**	**4.2-406**	ONC(=O)CCCCCCC(=O)NC1=CC=CC(=C1)C2=CN=N[N]2CC(O)C3=CC(=CC=C3)Br
**2-55895**	**600-4000**	ONC(=O)CCCC1=CC=CC=C1	**20-488747**	**18-433**	ONC(=O)CCCCCCC(=O)NC1=CC=CC(=C1)C2=CN=N[N]2CC3=CC=CC=C3
**3-138626**	**100000**	CC(=O)N(O)CCCCCCCC(=O)NC1=CC=CC=C1	**21-507592**	**17000-[illegible]**	ONC(=O)C1=NOC(=C1)CCCCC(=O)NC2CCCC2
**4-139193**	**4000-11000**	ON(CCCCCCC(=O)NC1=CC=CC=C1)C=O	**22-511749**	**190-6000**	CC(C)(C)OC(=O)NC1=CC=C(C=C1)C2=CC(=NO2)C(=O)NCCCCCCC(=O)NO
**5-141082**	**6800-**	ON(CCCCCC(=O)NC1=CC=CC=C1)C=O	**23-551151**	**1700**	ONC(=O)CCC1=CC=C(C=C1)C2=CC=C(O)C=C2
**6-146250**	**1.6-57**	C\C=C1/NC(=O)C(CS)NC(=O)C(NC(=O)CC(OC(=O)C(NC1=O)C(C)C)\C=C\CCS)C(C)C	**24-564916**	**260-74000**	NC1=CC=CC=C1NC(=O)CCCCCC(C2=C[NH]C3=C2C=CC=C3)C4=C[NH]C5=C4C=CC=C5
**7-152162**	**2350-2720**	ONC(=O)CCC1=CC=CC=C1	**25-1076939**	**3.0-5.9**	COC1=C(F)C=CC(=C1)[N]2C=C(N=N2)C3=CC=CC(=C3)NC(=O)CCCCCCC(=O)NO
**8-216641**	**0.8-400**	COC1=CC2=C([NH]C(=C2CC(=O)NC(CCCCCC(=O)NO)C(=O)NCCC3=C([NH]C4=C3C=CC=C4)C5=CC=CC=C5)C)C=C1	**26-1083439**	**53-200**	ONC(=O)CCCCCNC(=O)NC(=O)C1=CC=CC=C1
**9-217651**	**55-3000**	CN1CCCCC1C(=O)NC(CCCCCC(C)=O)C(=O)NC2=NC(=CS2)C3=CC=CC=C3	**27-1088734**	**28-5000**	ONC(=O)CCCCCCC(=O)NC1=CC(=CC=C1)C2=C[N](N=N2)C3=CC=C(C=C3)C(F)(F)F
**10-236060**	**2000-20000**	NC1=CC=CC=C1NC(=O)C2=CC3=C(C=C2)N=CC=N3	**28-1093033**	**7.5-217**	CC(C)(C)C1=C(C2CCCN(C2)CC3=CC=C(\C=C\C(=O)NO)C=C3F)C4=C([NH]1)C=CC=C4
**11-242533**	**31-50**	ONC(=O)\C=C\C1=CC=CC(=C1)C(=O)C2=CC3=C([NH]2)C=CC=C3	**29-1093041**	**27-35**	CCC1=CC(=CC=C1)NC(=O)CCCCC(=O)NO
**12-405072**	**2400-50000**	NC1=C(NC(=O)C2=CC=CC=C2)C=CC=C1	**30-1094108**	**3100-7200**	CC1=CC=CC(=C1)C2=CC(=C(N)C=C2)NC(=O)C3=CC=CC=C3
**13-440018**	**1400-19000**	CN(C)C1=CC=C(C=C1)C(=O)NCCCCCCNC(=O)CS	**31-1096398**	**10000**	NC1=C(NC(=O)C2=CC=CC=C2)C=C(C=C1)C3=CC4=C(S3)C=CC=C4
**14-466031**	**30000**	OCC1=NOC(=C1)CCCCC(=O)NC2=NC(=CS2)C3=CC=CC=C3	**32-1096982**	**11000-23000**	OC(=O)CC\C=C/CC1NC(=O)C2CCCN2C(=O)C(CC3=CC=CC=C3)NC(=O)C(CC4=C[NH]C5=C4C=CC=C5)NC1=O
**15-469134**	**30-430**	ONC(=O)C1=NOC(=C1)CCCCC(=O)NC2=NC(=CS2)C3=CC=CC=C3	**33-1097744**	**21000**	NC1=C(NC(=O)C2=CC=CC=C2)C=C(C=C1)C3=CC(=CC=C3)[N+]([O-])=O
**16-469275**	**83-5000**	CC(C)(C)OC(=O)NC1=CC(=CC=C1)C2=CC(=NO2)C(=O)NCCCCC(=O)NO	**34-1630109**	**35-630**	ONC(=O)\C=C\C1=CC(=CC=C1)C2=CC=C3N=CN=C(NC4=CC(=C(OCC5=CC(=CC=C5)F)C=C4)Cl)C3=C2
**17-470843**	**127-13200**	Cl.ONC(=O)\C=C\C1=CC=C(C=C1)C2=NOC(=C2)CN3CCOCC3	**35-1631915**	**89-382**	COC1=CC2=CC(=C1)\C=C/COC(CCCCCC(=O)NO)C(=O)NC3=CC=CC=C3OC2
**18-483693**	**7.9-1900**	ONC(=O)CCCCCCC(=O)NC1=CC=CC(=C1)C2=C[N](CC3=CC=CC=C3)N=N2	**36-1631916**	**204-700**	COC1=CC2=CC(=C1)CCCOC(CCCCCC(=O)NO)C(=O)NC3=CC=CC=C3OC2

Continued

ID	IC_{50}	SMILE code	ID	IC_{50}	SMILE code
**37-1722433**	**100000**	CC1=CC(=NC(=N1)SCC(=O)NCCC2=CC=CC=C2)O	**56-2170019**	**180-700**	CCC(=O)CCCCCC1NC(=O)C(CCCNC(N)=N)NC(=O)CCN(CC(C)C)C(=O)CN(CCC2=C[NH]C3=C2C=CC=C3)C1=O
**38-1767030**	**65000**	CNC(=O)\C(CCCCCCC(=O)NC1=CC2=CC=CC=C2C=C1)=N/O	**57-2323286**	**31-224**	CC(C)C1NC(=O)C[N]2N=NC(=N2)C3=CSC(=N3)CNC(=O)CC(OC1=O)\C=C\CCS
**39-1767033**	**10000**	CNC(=O)\C(CCCCCCC(=O)NC1=CC=C(C=C1)N(C)C)=N/O	**58-2323288**	**649-3270**	CC(C)C1NC(=O)C[N]2N=NN=C2C3=CSC(=N3)CNC(=O)CC(OC1=O)\C=C\CCS
**40-1767038**	**76000**	CNC(=O)\C(CCCCCCC(=O)NC1=CC(=CC=C1)C2=CN=CC=C2)=N/O	**59-2323289**	**14000-32000**	CCCCCCCC(=O)SCC\C=C\C1CC(=O)NCC2=NC(=CS2)C3=C[N](CC(=O)NC(C(C)C)C(=O)O1)N=N3
**41-1767039**	**1900-69000**	CNC(=O)\C(CCCCCCC(=O)NC1=CC(=CC=C1)C2=CN=C(Cl)C=C2)=N/O	**60-2333345**	**300-33000**	ONC(=O)C1CCCCC1
**42-1767042**	**5100-100000**	CNC(=O)\C(CCCCCCNC(=O)C1=NOC(=C1)C2=CC=C(NC(=O)OC(C)(C)C)C=C2)=N\O	**61-2347006**	**64-468**	CCC(C)C(=O)OC1CC(C)C=C2C=CC(C)C(CCC(O)CC(O)CC(=O)NO)C12
**43-1800241**	**15000-23000**	CC(=O)NC1=CC=C(C=C1)C(=O)CSC2=NC(=CC(=N2)C)O	**62-2381517**	**120-2300**	ONC(=O)C1=CC(=CC=C1)C(=O)NCCC2=CC=CC=C2
**44-1800244**	**100000**	CC(=O)NC1=CC=C(C=C1)C(=O)CSC2=NC(=CC(=N2)C)C	**63-2407721**	**410-12000**	NC1=CC=CC=C1NC(=O)C2=CC=C(CNC3=NC(CS3)CC4=CC=CC=C4)C=C2
**45-1800247**	**100000**	CC(=O)NC1=CC=C(C=C1)C(=O)COC2=CC(=CC(=N2)O)C	**64-2408778**	**230-2311**	NC1=C2C(=NC=N1)[N](N=C2C3=CC=C(Cl)C=C3)C4=CC=CC(=C4)C5=CN=N[N]5CCCCCC(=O)NO
**46-1800248**	**100000**	CC(=O)NC1=CC=C(CSC2=NC(=CC(=N2)O)C)C=C1	**65-2414098**	**6-282**	ONC(=O)CCCCCCC1=NC2=CC=C(Br)C=C2[NH]1
**47-1800377**	**100000**	CC1=CC(=NC(=N1)SCC(=O)C2=CC3=C(OCCO3)C=C2)O	**66-2417782**	**21-2580**	CC(C)(C)OC(=O)NC1=CC=CC(=C1)C2=CSC(=N2)NC(=O)CCCCCCC(=O)NO
**48-1800379**	**100000**	CC1=CC(=NC(=N1)SCC(=O)C2=CC=CS2)O	**67-2417783**	**5000-28000**	CC1=C(C(=O)NCCCCCNC(=O)CS)C(=NO1)C2=CC=CC=C2
**49-1914702**	**45-780**	ONC(=O)CCCCCCNC(=O)C1=NOC(=C1)C2=CC=C(NC(=O)C3=CC(=CC(=C3)N=[N+]=[N-])CN=[N+]=[N-])C=C2	**68-2425958**	**1700-6400**	CC1=NC2=CC(=CC=C2C(=O)N1CCC3=CC=CC=C3)\C=C\C(=O)NO
**50-1938433**	**65-1760**	ONC(=O)\C=C\C1=CC2=C(OC3(CCN(CC3)CC4=CC=CC=C4)CC2=O)C=C1	**69-2431906**	**1300-50000**	C[N]1C=CC=C1C(=O)N2CC3=C(C2)C=C(C=C3)C(=O)NO
**51-2022829**	**5.2-7.6**	O=C(NC(CCCCCCSSC1=CC=CC=N1)C(=O)OCC2C3=C(C=CC=C3)C4=C2C=CC=C4)OCC5=CC=CC=C5	**70-2436595**	**20-1200**	CC(C)C1NC(=O)C2(C)CSC(=N2)C3=COC(=N3)CNC(=O)CC(OC1=O)\C=C\CCS
**52-2048749**	**124-361**	COC1=CC=C(C=C1)[S](=O)(=O)[N]2C=CC3=C2C=CC(=C3)\C=C\C(=O)NO	**71-2442698**	**140-230**	COC1=CC(=CC(=C1O)OC)C2C3C(COC3=O)C(NC4=CC=C(NC(=O)CCCCCCC(=O)NO)C=C4)C5=CC6=C(OCO6)C=C25
**53-2048750**	**2.3-18**	COC1=C(OC)C=C(C=C1)[S](=O)(=O)[N]2C=CC3=C2C=CC(=C3)\C=C\C(=O)NO	**72-2448576**	**18-10000**	CCC(=O)CCCCCC(NC(=O)C1CN(C)C1)C2=NC=C([NH]2)C3=CC4=CC=CC=C4N=C3OC
**54-2057821**	**350-7000**	NC1=C(NC(=O)C2=CC=C(CNC(=O)OCC3=CC(=CC(=C3)N=[N+]=[N-])CN=[N+]=[N-	**73-3098695**	**34-89**	ONC(=O)CCCCCC(NC(=O)C1CC(=O)N1)C(=O)NC2=CC=CC=C2
**55-2057828**	**3000-1800**	NC1=C(NC(=O)C2=CC(=C(OCCN=[N+]=[N-])C=C2)N=[N+]=[N-])C=C(C=C1)C3=CC=CS3	**74-3098697**	**1400-5000**	ONC(=O)CCCCCC(NC(=O)C1CCCC(=O)N1)C(=O)NC2CCCC2

Table D Binding energy values (kcal/mol) of training set compounds

Inhibitors	Binding energies			
	HDAC1	HDAC2	HDAC3	HDAC4
**MS-275**	-13.1	-13.9	-9.42	-9.4
**LBH-589**	-18.5	-29.58	-13.55	-20.21
**LAQ-824**	-12.48	-30.32	-13.2	-17.74
**TSA**	-13.57	-20.75	-9.1	-21.87
**SAHA**	-10.65	-21.64	-12.7	-13.34
**Belinostat**	-22.37	-24.83	-16.4	-23.92
**Oxamflatin**	-12.23	-20.34	-10.1	-21.22
**Pyroxamide**	-11.52	-21.69	-14.6	-14.61
**Mocetinostat**	-24.15	-21.8	-12.23	-
**Scriptaid**	-9.5	-17.85	-19.26	-12.47

Table E Molecular descriptors of training set compounds

Compounds	Mol. Weight	Mol. Polarizability	Huckel Analysis (pi energy)	Molar Refractivity	LogD	LogP	TPSA	HBD	HBA
**MS-275**	376	40.19	45.8	108.288	2	2.31	106.34	4	7
**LBH-589**	349	41.08	38.42	105.499	1	2.39	77.15	4	5
**LAQ-824**	379	43.6	42.6	111.936	1	3.18	88.59	4	5
**TSA**	274	29.44	30.57	80.511	1.4	2.41	92.42	4	5
**Saha**	264	28.1	25.62	73.805	1.67	2.46	78.43	3	5
**Belinostat**	318	32.39	39.69	83.47	1.5	1.81	95.5	3	6
**Oxamflatin**	344	35.82	42.23	93.798	2	2.73	95.5	3	6
**Pyroxamide**	265	27.23	26.23	71.648	1	1.396	91.32	3	6
**Mocetinostat**	396	44.93	47.6	120.316	2.6	2.93	105.82	4	7
**Scriptaid**	326	35.05	35.65	100.662	1	-3.9	92.47	2	6

Continued

SASA	Vander Wall's Volume	Weiner Index (W)	Platt Index	Randic Index	Balaban Index	Harary Index	Szeged Index
528.78	336.84	2622	78	13.61	1.18	98.96	3678
516.32	325.14	2128	74	12.67	1.27	89.6	2888
557.56	351.84	2462	86	14.53	1.27	100.51	3306
390.46	255.43	950	52	9.43	2.28	61.08	1142
422.78	253	954	44	9.22	1.8	53.35	1098
413.55	265.6	1160	62	10.51	1.65	72.9	1622
445.67	292.02	1667	66	11.51	1.55	79.64	2285
416.05	249.8	954	44	9.22	1.8	53.35	1098
546.41	351.37	3031	88	14.69	1.03	112.36	4662
452.79	288.37	1432	72	11.63	1.42	85.84	2428

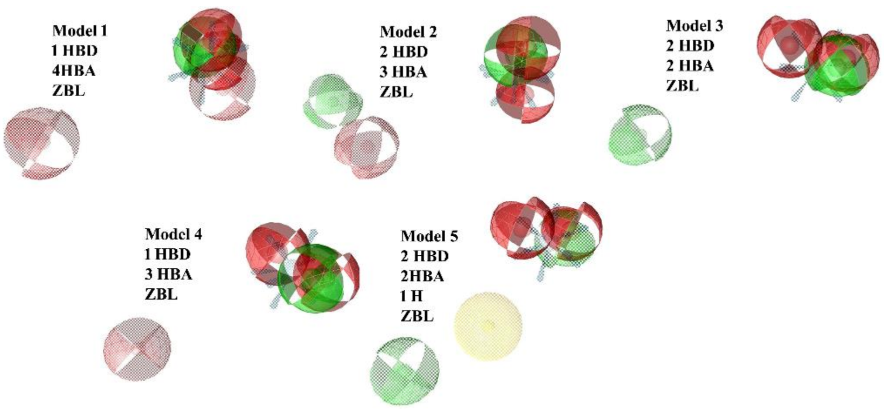

Fig A Top five pharmacophore models. HBD, Hydrogen Bond Donor (green); HBA, Hydrogen Bond Acceptor (red); H, Hydrophobic site (yellow); ZBL, Zinc Binding Location (blue)

Table F pIC_{50} and average pIC_{50} values of training set compounds

**Compound**	**pIC_{50} values (nm)**				**Avg. pIC_{50}**
	HDAC1	HDAC2	HDAC3	HDAC8	
**MS-275**	6.92	6.60	6.92	***	6.81
**LBH-589**	6.60	6.60	6.60	6.60	6.60
**LAQ-824**	8.49	7.80	7.97	8.41	8.16
**TSA**	8.30	7.74	8.69	7.60	8.07
**SAHA**	7.46	6.69	7	7	7.03
**Belinostat**	7.74	***	7.33	***	7.53
**Oxamflatin**	7	6.63	6.63	6.63	6.72
**Pyroxamide**	6.70	6.70	6.70	6.70	6.70
**Mocetinostat**	6.82	6.53	6.21	***	6.51
**Scriptaid**	6.69	6.15	7	6.52	6.58

Table G Screening results of pharmacophore models for validation

Model No.	True Positive (TP)	False Positive (FP)	True Negative (TN)	False Negative (FN)	Ha (active retrieved)	Ht (total retrieved)
**Model-1**	37	10	21	9	37	47
**Model-2**	24	10	20	8	24	34
**Model-3**	27	9	21	14	27	36
**Model-4**	22	14	16	19	22	36
**Model-5**	20	8	22	21	20	28

Formulas for sensitivity and specificity:

$$Sensitivity = \frac{TP}{TP+FN}$$

$$Specificity = \frac{TN}{TN+FP}$$

Whereas
$TP = True\ Positives$
$FN = False\ Negative$
$TN = True\ Negative$
$FP = False\ Positive$

Formulas for %Y, E.F and G.H:

$$\%Y = \left(\frac{Ha}{Ht}\right) * 100$$

$$E.F = \frac{(Ha/Ht)}{(A/D)}$$

$$GH = \frac{\{Ha\,(3A+Ht)\}}{(4*Ht*A)} * 1 - \frac{(Ht-Ha)}{(D-A)}$$

Whereas
$\%Y = Percent\ Yeild$
$Ha = Total\ actives\ retreived$
$Ht = Total\ hits\ retrieved$
$A = Total\ actives\ in\ dataset$
$D = Total\ decoys\ in\ dataset$

Formula for pIC_{50}:

$$pIC_{50} = -\log IC50\,(M)$$

Formula for Geometric Mean:

$$Geometric\ Mean = \left(\prod_{k=1}^{n} A_k\right)^{\frac{1}{n}} = \sqrt[n]{A_1 A_2 \ldots\ldots A_n}$$

Where
$n = Total\ number\ of\ IC50\ values\ for\ each\ compound$
$A = Each\ IC_{50}\ value$

ANoVA represents hypothesis testing where the null and alternate hypothesis are:

$$H_\circ: \beta_i = 0 \quad for\ all\ i, i = 1, \ldots\ldots, n$$

$$H_1: \beta_i \neq 0 \quad for\ atleast\ 1\ coefficient$$

Where
$H_\circ$ = Null hypothesis
H_1 = Alternate hypothesis

Table H Binding energies of 16 novel hits compounds with class 1 HDACs

Inhibitors	Binding Energies (kcal/mol)			
	HDAC1	HDAC2	HDAC3	HDAC8
**1**	-14	-21.87	-10	-24
**2**	-10	-16	-6	-9
**3**	-13	-23.72	-18	-25
**4**	-10	-22.35	-14	-21.8
**5**	-10	-16	-10	-14
**6**	-11	-22	-13	-14.7
**7**	-10	-23.62	-17	-23.7
**8**	-17	-24	-14	-21.4
**9**	-21	-29.26	-22	-18
**10**	-16	-23.33	-16	-18.3
**11**	-16	-23	-16	-18
**12**	-11	-22	-16	-23.1
**13**	-20	-25.15	-22	-23.6
**14**	-20	-24.56	-21	-26
**15**	-15	-24	-10	-19.3
**16**	-14	-25.58	-13	-23.8

Table I Molecular descriptors for 10 selected lead-like compounds

	Mol. Weight	N	Mol. Polariza-bility	Huckel Analysis (pi energy)	Molar Refracti-vity	LogD	LogP	TPSA	(nOHNH)	(nON)	SASA	Vander Wall's Volume
**C1**	361.29	6	32.93	53.19	87.88	2.6	2.56	87.85	2	6	439.11	287.38
**C2**	385.36	7	38.46	50.97	100.8	1.29	1.63	106.31	2	8	528.36	329.62
**C3**	261.06	4	25.1	33.5	65.54	0.2	0.61	95.69	2	6	335.39	218.52
**C4**	377.29	5	32.25	53.54	89.73	1.8	1.9	110	2	8	463.5	294.9
**C5**	298.3	3	30.25	39.28	101.55	3.4	1.4	80.15	2	6	379.51	250.87
**C6**	274.7	3	27.8	33.77	75.02	2.8	3.3	61.69	2	4	346.05	227.67
**C7**	355.1	6	35.91	46.65	94.34	1.45	1.7	97.08	2	4	480.67	303.33
**C8**	360.4	5	36.8	43.98	91.985	1.2	1.8	111.63	3	7	485.46	298.5
**C9**	431.2	5	36.05	40.64	92.46	-0.2	-0.12	138.67	4	9	475.7	307.86
**C10**	309.3	4	32.77	38.09	84.65	2.2	2.9	78.62	2	5	417.63	270.02

Continued

Weiner Index (W)	Platt Index	Randic Index	Balaban Index	Harary Index	Szeged Index
**1808**	132	17.72	1.53	94.77	2565
**1651**	76	11.94	1.33	89.34	2404
**748**	56	8.97	1.67	60.24	1268
**2030**	84	12.76	1.43	100.4	2740
**1235**	64	1.72	1.34	72.57	1930
**838**	52	9.15	1.43	57.32	1186
**1847**	78	12.51	1.5	94.43	2704
**1719**	78	11.88	1.25	88.67	2416
**1710**	76	11.57	1.62	87.73	2388
**1200**	70	11.06	1.56	81.88	2086

N= No. of rotatable bonds

TPSA= Topological Polar Surface Area

nOHNH= Hydrogen Bonds Donor

nON= Hydrogen Bond Acceptors

SASA= Solvent Accessible Surface Area

Table J IUPAC names and SMILE codes for compound C1-C10

Compound	IUPAC name	SMILE code
**C1**	2-{[(2Z)-2-{[2-(difluoromethoxy)phenyl]methylidene}-3-oxo-2,3-dihydro-1-benzofuran-6-yl]oxy}acetamide	NC(=O)COC1=CC=C2C(=O)\C(OC2=C1)=C\C3=C(OC(F)F)C=CC=C3
**C2**	(3-{[(2Z)-6-(carbamoylmethoxy)-3-oxo-2,3-dihydro-1-benzofuran-2-ylidene]methyl}phenyl)azinic acid	NC(=O)COC1=CC=C2C(=O)\C(OC2=C1)=C\C3=CC(=CC=C3)[NH+](O)[O-]
**C3**	2-[(3-acetyl-2-oxo-2H-chromen-7-yl)oxy]acetamide	CC(=O)C1=CC2=C(OC1=O)C=C(OCC(N)=O)C=C2
**C4**	({1,7-dimethyl-4-oxo-3H,4H-furo[3,4-d]pyridazin-5-yl}carbamoyl)methyl 3,5-difluorobenzoate	CC1=C2C(=NNC(=O)C2=C(NC(=O)COC(=O)C3=CC(=CC(=C3)F)F)O1)C
**C5**	N'-[(1E)-1,4-benzodioxin-6-ylmethylidene]-3-hydroxybenzohydrazide	OC1=CC=CC(=C1)C(=O)N\N=C\C2=CC3=C(O[CH2]=[CH2]O3)C=C2
**C6**	N'-[(1E)-(4-chlorophenyl)methylidene]-3-hydroxybenzohydrazide	OC1=CC=CC(=C1)C(=O)N\N=C\C2=CC=C(Cl)C=C2

**C7**	2-{[(2Z)-2-[(3,4-dimethoxyphenyl)methylidene]-3-oxo-2,3-dihydro-1-benzofuran-6-yl]oxy}acetamide	COC1=C(OC)C=C(C=C1)\C=C2/OC3=CC(=CC=C3C2=O)OCC(N)=O
**C8**	7-methoxy-N-[(4-sulfamoylphenyl)methyl]-1-benzofuran-2-carboxamide	COC1=C2OC(=CC2=CC=C1)C(=O)NCC3=CC=C(C=C3)[S](N)(=O)=O
**C9**	2-(5-bromo-2,4-dioxo-1,2,3,4-tetrahydropyrimidin-1-yl)-N-[1-(4-sulfamoylphenyl)ethyl]acetamide	CC(NC(=O)CN1C=C(Br)C(=O)NC1=O)C2=CC=C(C=C2)[S](N)(=O)=O
**C10**	2-[(4-methyl-2-oxo-3-phenyl-2H-chromen-6-yl)oxy]acetamide	CC1=C(C(=O)OC2=C1C=C(OCC(N)=O)C=C2)C3=CC=CC=C3

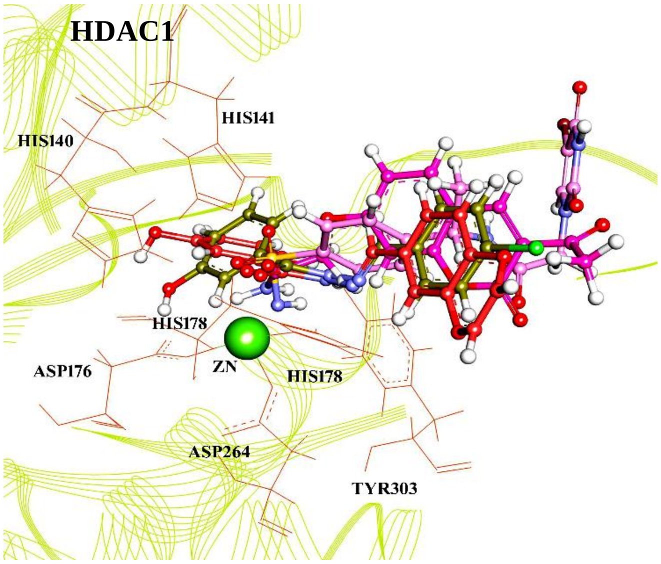

Fig B Binding view of selected compounds with HDAC1. A combined 3D binding view of C3, C5, C6 and C9 compounds with HDAC1. These compounds form hydrogen bonds with HIS140, HIS141, ASP176, HIS178, ASP264 and TYR303 and Zn+2 of HDAC1

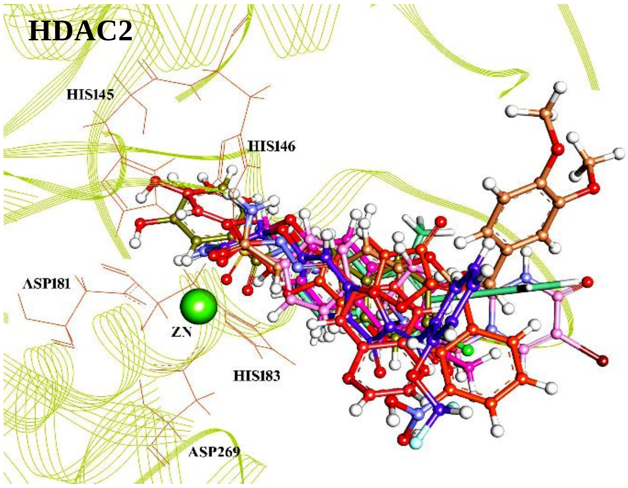

Fig C Combined 3D binding view of C1, C2, C3, C5, C6, C7, C9 and C10 compounds with HDAC2. These compounds formed hydrogen bonds with HIS145, HIS146, ASP181, HIS183, ASP269 and Zn+2 of HDAC2

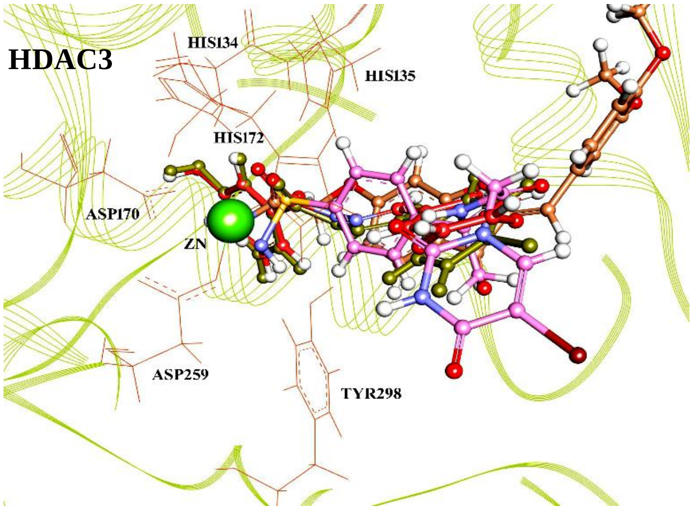

Fig D Binding view of selected compounds with HDAC3. Combined 3D binding view of C5, C6, C7 and C9 compounds with HDAC3. These compounds formed hydrogen bonds with HIS134, HIS135, ASP170, HIS172, ASP259, TYR298 and Zn+2 of HDAC3

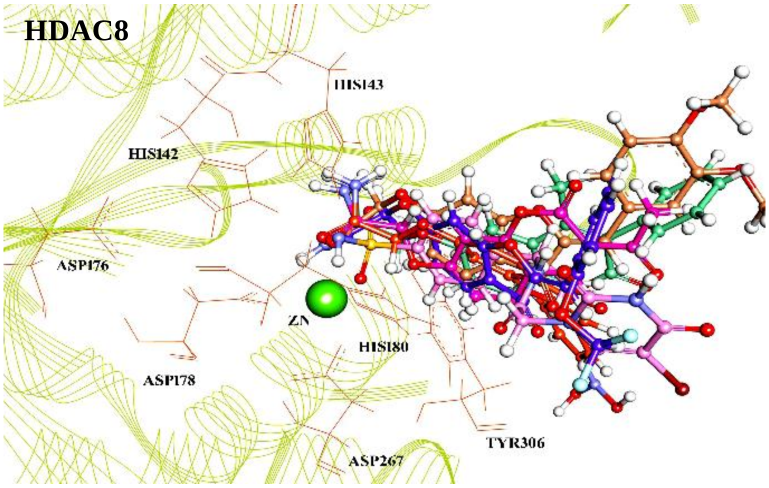

Fig E Binding view of selected compounds with HDAC8. Combined 3D binding view of compound C1, C2, C3, C7, C9 and C10 with HDAC8. These compounds formed hydrogen bonds with HIS142, HIS143, ASP178, HIS180, ASP267, TYR306 and Zn+2 of HDAC8.

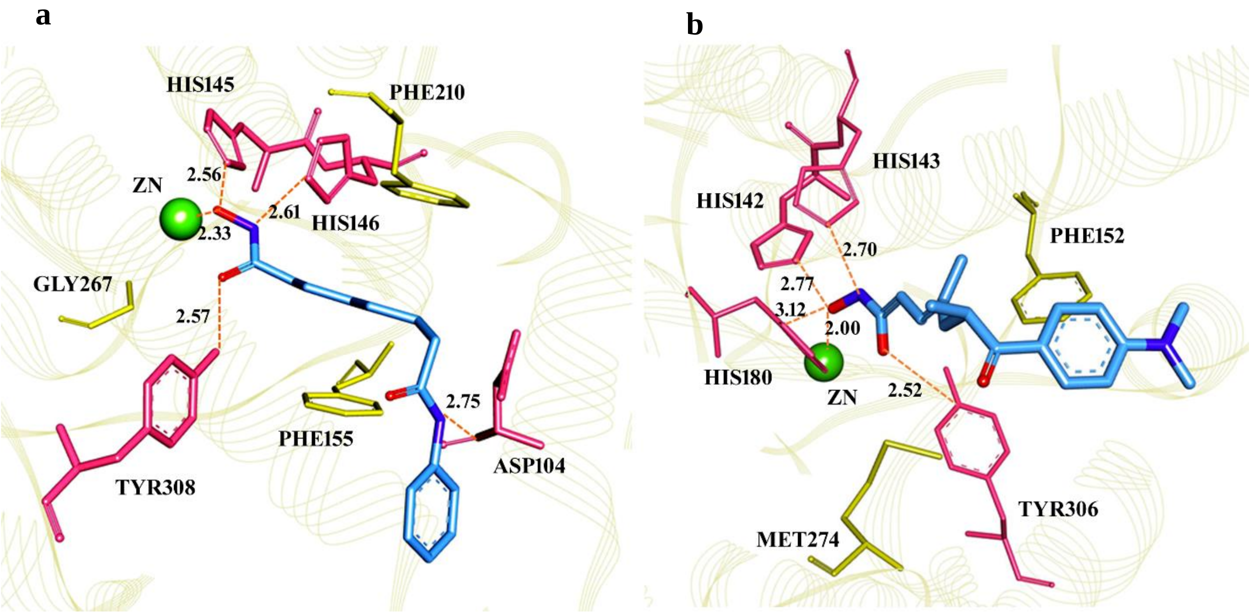

Fig F Experimental 3D binding view of **(a)** compound SAHA with HDAC2 and **(b)** compound Trichostatin A with HDAC8. Residues shown in pink formed hydrogen bonds with inhibitors. Hydrophobic residues and Zn^{+2} are shown in yellow and green, respectively.

HDAC2

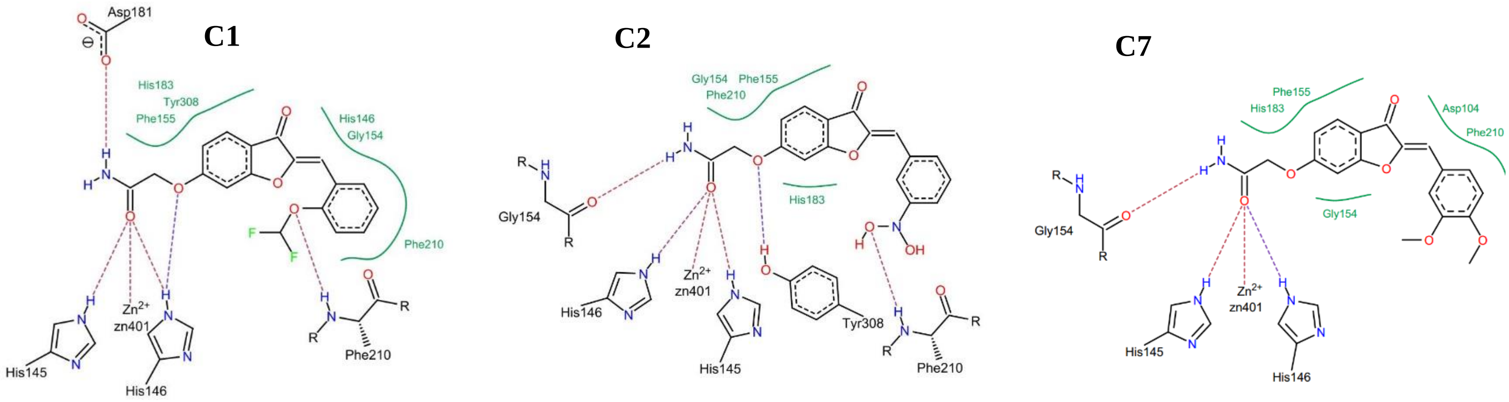

Fig G 2D binding view of C1, C2 and C7 with HDAC2.

HDAC8

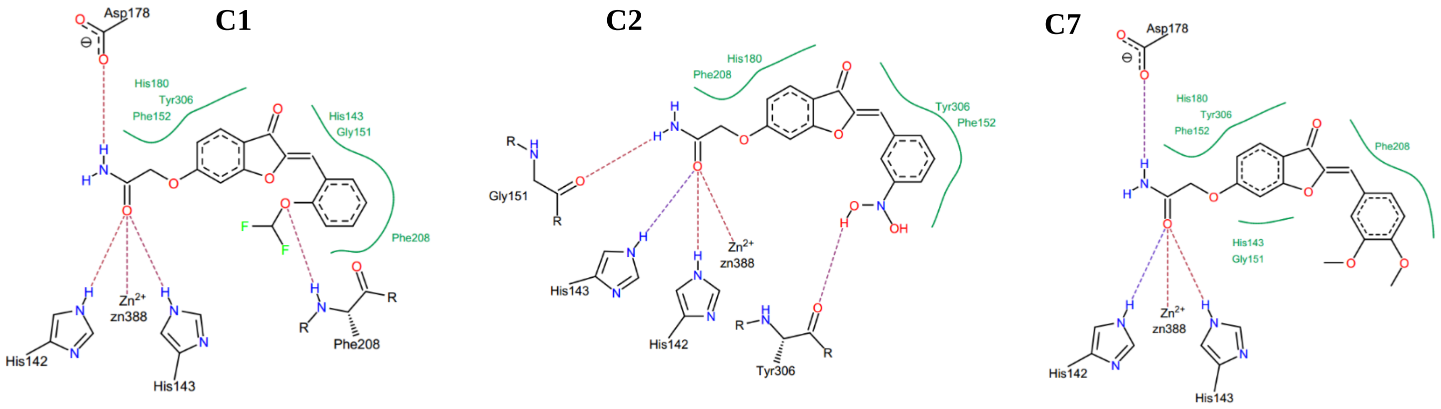

Fig H 2D binding view of C1, C2 and C7 with HDAC8.

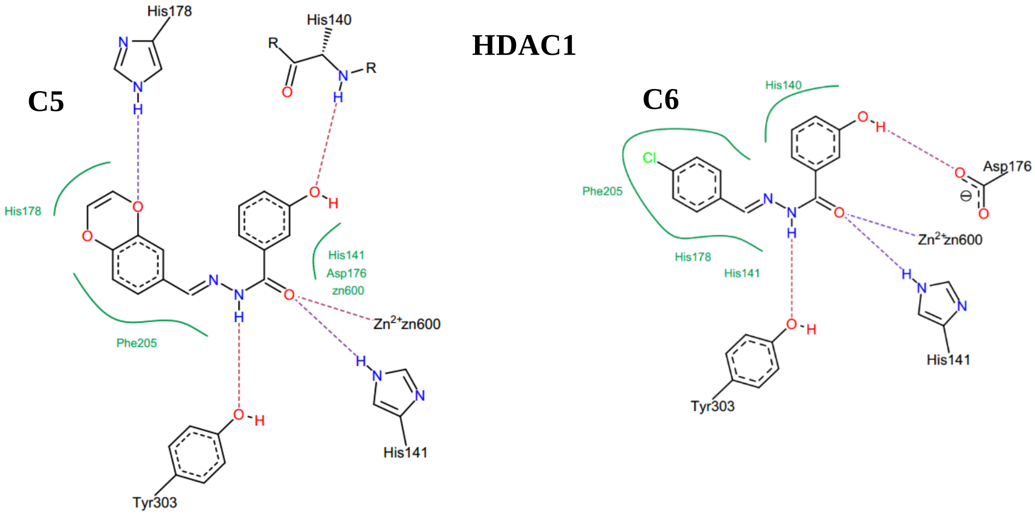

Fig I 2D binding view of C5 and C6 with HDAC1.

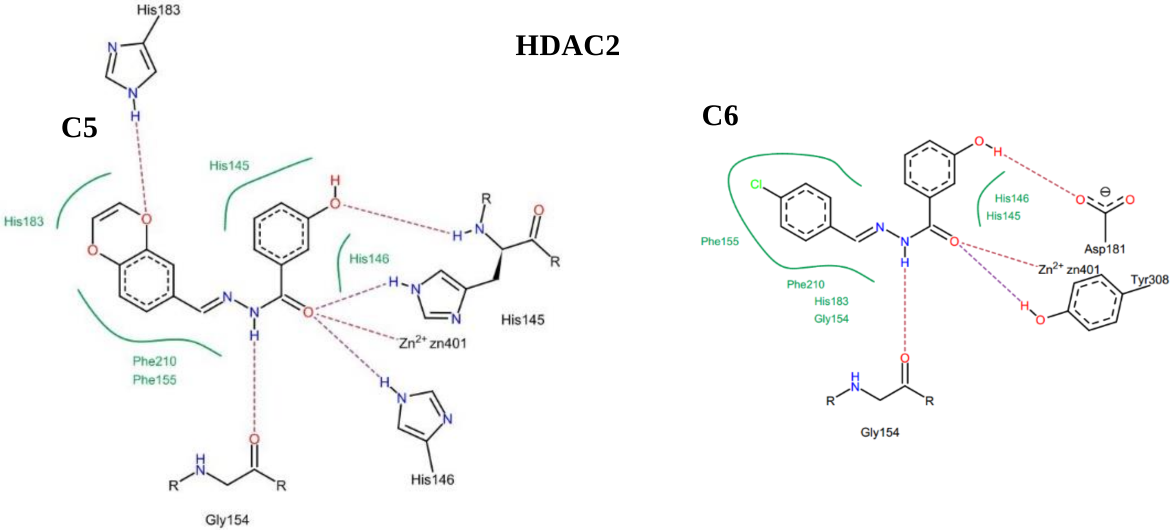

Fig J 2D binding view of C5 and C6 with HDAC2.

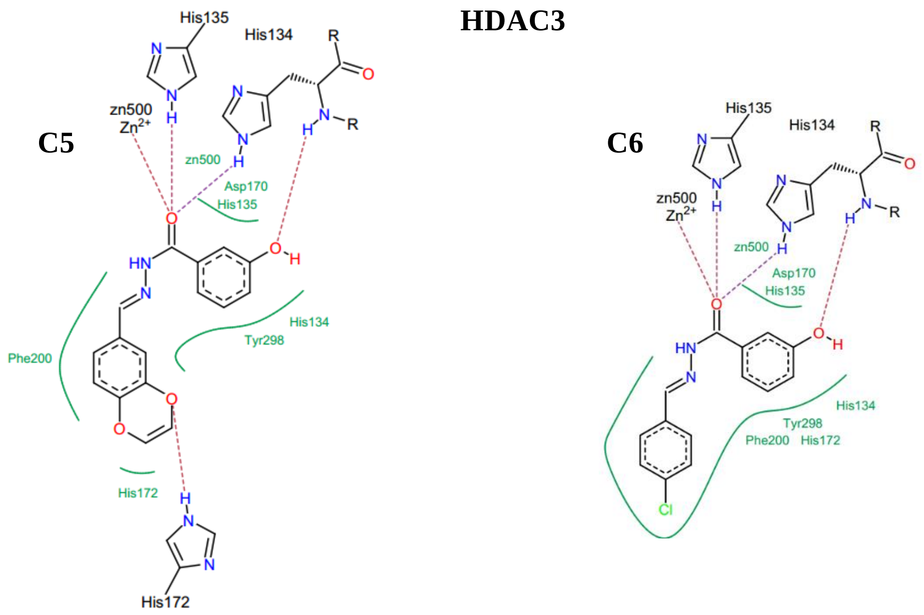

Fig K 2D binding view of C5 and C6 with HDAC3.
